# Structural determinants of inverted Alu-mediated backsplicing revealed by -MaP and -JuMP

**DOI:** 10.1093/nar/gkaf433

**Published:** 2025-05-21

**Authors:** Justin M Waldern, Colin Taylor, Catherine A Giannetti, Patrick S Irving, Scott R Allen, Mingyi Zhu, Rolf Backofen, David H Mathews, Kevin M Weeks, Alain Laederach

**Affiliations:** Department of Biology, University of North Carolina at Chapel Hill, Chapel Hill, NC 27599, United States; Department of Biology, University of North Carolina at Chapel Hill, Chapel Hill, NC 27599, United States; Department of Chemistry, University of North Carolina, Chapel Hill, NC 27599, United States; Department of Chemistry, University of North Carolina, Chapel Hill, NC 27599, United States; Department of Biology, University of North Carolina at Chapel Hill, Chapel Hill, NC 27599, United States; Department of Biochemistry & Biophysics and Center for RNA Biology, University of Rochester Medical Center, Rochester, NY 14526, United States; Bioinformatics Group, Department of Computer Science, University of Freiburg, 79110 Freiburg, Germany; Signaling Research Centers BIOSS and CIBSS, University of Freiburg, 79110 Freiburg, Germany; Department of Biochemistry & Biophysics and Center for RNA Biology, University of Rochester Medical Center, Rochester, NY 14526, United States; Department of Chemistry, University of North Carolina, Chapel Hill, NC 27599, United States; Department of Biology, University of North Carolina at Chapel Hill, Chapel Hill, NC 27599, United States

## Abstract

Biogenesis of circular RNA usually involves a backsplicing reaction where the downstream donor site is ligated to the upstream acceptor site by the spliceosome. For this reaction to occur, these sites must be in proximity. Inverted repeat sequences, such as Alu elements, if positioned in the upstream and downstream introns, can base pair and represent one mechanism for inducing proximity. Here, we investigate the pre-mRNA structure of the human *HIPK3* gene at exon 2, which forms a circular RNA via backsplicing. We leverage multiple chemical probing approaches, including the recently developed SHAPE-JuMP (selective 2′-hydroxyl acylation analyzed by primer extension and juxtaposed merged pairs) strategy, to characterize secondary and tertiary interactions in the pre-mRNA that govern backsplicing. Our data confirm that the antisense Alu elements AluSz(−) and AluSq2(+), in the upstream and downstream introns, form a highly paired interaction. Circularization requires formation of long-range Alu-mediated base pairs but does not require the full-length AluSq2(+). In addition to confirming long-range base pairs, our SHAPE-JuMP data identified multiple long-range interactions between non-pairing nucleotides. Genome-wide analysis of inverted repeats flanking circular RNAs confirms that the presence of these elements favors circularization, but with modest predictive power. Together, our study suggests that secondary structure considerations alone do not fully explain backsplicing and that additional interactions are involved.

## Graphical Abstract

**Graphical Abstract:**
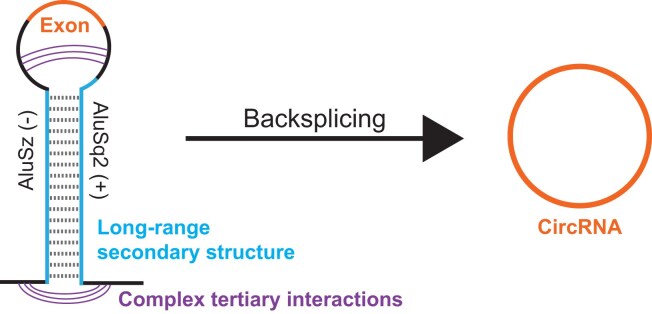

## Introduction

Circular RNAs (circRNAs) are formed by a noncanonical splicing process termed backsplicing [1–3]. Backsplicing is a spliceosome-catalyzed process where a downstream splice donor (5′ splice site) is linked to an upstream splice acceptor (3′ splice site), covalently joining together one or more exons in a 5′ to 3′ phosphodiester linkage to form a circle [4, 5]. CircRNAs are often flanked by intronic inverted repeats, which have been proposed to drive backsplicing based on inter-intron RNA base pairing via sequence complementarity [2, 3]. Inverted repeats were first implicated in backsplicing over 30 years ago in a circRNA from the mouse *Sry* gene [6]. In humans, 88% of circRNAs are flanked by intronic Alu elements [7]. Alu elements are non-autonomous retrotransposons that comprise ∼10% of the human genome [8]. Although Alu elements can be categorized into distinct familial lineages, the sequence similarity between any two Alu elements is typically >80% [9]. The high degree of sequence similarity between Alu elements leads to a high degree of complementarity between inverted repeat Alu elements, which can base pair to form double-stranded RNA as evidenced by high degrees of ADAR editing in inverted repeat Alu elements in close genomic proximity [10, 11]. The long-range Alu–Alu interaction induced by a novel Alu insertion has been proposed to cause an exon-skipping event responsible for tail loss during the evolution of humans [12]. Others have also shown that novel Alu insertions can alter splicing and potentially cause disease through mis-splicing [13]. A leading hypothetical mechanism for backsplicing is that sense and antisense Alu elements, as inverted repeats, form inter-intronic structure across exons and drive backsplicing via sequence complementarity and long-range RNA structure that bring splice sites into proximity in three-dimensional space [3, 14].

The human homeodomain interacting protein kinase 3 (*HIPK3*) gene produces a single-exon circRNA from exon 2 that has been previously studied to decipher the sequence components required for backsplicing [2, 3, 15]. The *HIPK3* exon 2 circRNA is flanked by intronic sense and antisense Alu elements, AluSz(−) and AluSq2(+), which are essential for backsplicing [2]. However, regional deletions within these Alu elements can either ablate or retain backsplicing, suggesting that some portions of the Alu elements are essential for backsplicing whereas others are dispensable [2]. Furthermore, when applying *in silico* energy minimization to predict Alu element structures, the thermodynamic stability of the truncated hairpin is not predictive of backsplicing, suggesting that multiple structural conformations or more complex structural interactions beyond simple base pairing enable backsplicing [2]. Distance requirements for backsplicing suggest that there may be complex interactions at play. For example, moving the downstream AluSq2(+) up to 1500 nucleotides away from the exon does not affect backsplicing, whereas moving the upstream AluSz(−) only 500 nucleotides away from the exon ablates backsplicing [16]. Thus, based on sequence and predicted base-pairing considerations alone, it is difficult to predict whether a given set of Alu elements will favor backsplicing. Furthermore, certain RNA binding proteins, such as ADAR and DHX9, specifically act on long double-stranded RNAs, such as paired inverted repeat Alu elements, and regulate backsplicing by targeting these structures [17]. One aspect of pre-mRNA that remains understudied is its structure, and here we leverage novel chemical probing approaches to investigate the secondary and tertiary structures of the *HIPK3* pre-mRNA.

The size of human pre-mRNAs, where median intron lengths are over 1400 nucleotides [18], makes structural studies challenging. Chemical structure probing coupled with next-generation sequencing is one approach that can routinely probe structures of large RNAs [19, 20]. However, most chemical structure probing techniques detect whether a nucleotide is paired [21, 22] but do not directly identify the pairing partner. SHAPE-JuMP (selective 2′-hydroxyl acylation analyzed by primer extension and juxtaposed merged pairs) leverages a highly processive reverse transcriptase coupled with a bivalent cross-linking reagent to identify paired and proximal nucleotides [23, 24]. Here we leverage this strategy, coupled with conventional per-nucleotide chemical probing, to understand the structural determinants of backsplicing in the *HIPK3* circRNA. Integration of these data supports a complex structural model in which noncanonical base-pairing interactions in the pre-mRNA are as important in favoring backsplicing as inverted repeat base pairing. Taken together, our data begin to reveal unexplored three-dimensional features of pre-mRNA structure and its role in regulating splicing.

## Materials and methods

### Construct design

HIPK3 constructs were initially designed based on [2]. Constructs were synthesized by Genscript to insert the HIPK3 exon 2 and its surrounding sequence (GRCh38/hg38 chr11:33285723–33288525) into a pcDNA 3.1 backbone. Mutant derivatives of this construct [ΔAluSz(−), ΔAluSq2(+), ΔΔAlu, Δ157AluSq2(+), and Δ189AluSq2(+)] were also generated by Genscript and full sequence files can be found in Supplementary Files S1–S8.

### Cell culture and transfection

HeLa cells (ATCC number CCL 2) were cultured in high-glucose Dulbecco’s modified Eagle’s medium (DMEM; Gibco) supplemented with 10% fetal bovine serum (Sigma) and 0.5% pen/strep (Gibco). Cells were maintained at 37°C and 5% CO_2_.

Transfections were performed with Lipofectamine 3000 (Invitrogen) per manufacturer protocols. Cells were seeded 24 h prior to transfection, where 1 μg of plasmid was transfected per well in six-well plates. Medium was exchanged for fresh medium 24 h post-transfection, and cells were harvested 48 h post-transfection.

### RNA preparation (*in vitro*)

DNA template for *in vitro* transcription was generated with polymerase chain reaction (PCR; primers JW25 and JW15 in Supplementary File S9) using the construct plasmids as a template and Q5 Hot Start High-Fidelity DNA Polymerase (NEB). PCR reactions were cleaned up with the Monarch PCR & DNA Cleanup Kit (NEB), with product size and integrity verified by agarose gel electrophoresis. RNA was *in vitro* transcribed (IVT) from the clean PCR product using HiScribe T7 High Yield RNA Synthesis Kit (NEB), followed by TurboDNase treatment (Invitrogen). RNA was isolated by ethanol precipitation.

### Chemical probing—*in vitro* SHAPE

Selective 2′-hydroxyl acylation analyzed by primer extension and mutational profiling (SHAPE-MaP) with 5-nitroisatoic anhydride (5NIA) was used to probe RNA structure at all four nucleotides. To begin, 3 μg of IVT RNA was diluted in a total volume of 50 μl and denatured (65°C for 5 min, followed by ice for 2 min). Denatured RNA was combined with 50 μl of 2× Bicine folding buffer (600 mM Bicine, pH 8.3, 300 mM NaCl, 10 mM MgCl_2_) and 1 μl RNase inhibitor, and then refolded at 37°C for 10 min. RNA was then added to 10 μl of 250 mM 5NIA (in dimethyl sulfoxide (DMSO)) or 10 μl DMSO as a control and incubated at 37°C for an additional 10 min, until finally quenching on ice. RNA cleanup was performed with RNAClean XP beads (Beckman Coulter).

RNA was reverse transcribed under mutational profiling reverse transcription conditions (MaP-RT) with SuperScript II (Invitrogen), randomly primed with random nonamers. Briefly, 30 μl of RNA was premixed with 1 μl of 200 ng/μl Random Primer 9 (NEB) and 2 μl of 100 mM dNTPs (NEB) and heated at 65°C for 5 min, and then chilled on ice for 2 min. Added to each reaction was 4 μl of first strand buffer (0.5 M Tris, pH 8.0, 0.75 M KCl), 4 μl of 100 mM dithiothreitol (DTT), 0.48 μl of 100 mM MnCl_2_, 0.5 μl RNase inhibitor (NEB), and 2 μl SuperScript II. Reactions were carried out with the following program: 25°C for 2 min, 42°C for 3 h, and 70°C for 15 min. Reverse transcription reactions were cleaned up with G50 columns (Cytiva).

Second strand synthesis was performed with the NEBNext Ultra II Non-Directional RNA Second Strand Synthesis Module (NEB). Libraries were generated with the NEBNext Ultra II FS DNA Library Prep Kit for Illumina (NEB). Library concentration and purity were verified using the Qubit dsDNA HS kit (Invitrogen) and the Bioanalyzer 2100 (Agilent), followed by equimolar pooling and sequencing on the Illumina MiSeq platform.

### Chemical probing—*in vitro* DMS

For structure probing with dimethyl sulfate (DMS), 3 μg of IVT RNA was diluted in 15 μl of molecular biology grade water and heat denatured (65°C for 5 min, followed by ice for 2 min). Denatured RNA was combined with 15 μl of 2× Bicine folding buffer and refolded at 37°C for 30 min. Nine microliters of folded RNA was then added to either 1 μl of 1.7 M DMS (final concentration 170 mM) prepared in a 1:2 (v/v) nitromethane/sulfolane (NS) solution or 1 μl of NS solution as a vehicle control as in [25]. The reaction was incubated at 37°C for 6 min before quenching with an equal volume of 1:3 (v/v) beta-mercaptoethanol (BME) in water. Reactions were buffer exchanged with G50 spin columns (Cytiva) prior to MaP-RT.

DMS-optimized MaP-RT was performed as previously described [26]. Briefly, 8.8 μl of the probed RNA was combined with 2 μl of 10 mM dNTPs (NEB) and 1 μl of 200 ng/μl random nonamer (Random Primer 9, NEB). This mixture was heat denatured at 98°C for 1 min and then chilled on ice for 2 min. Added to each reaction was 2 μl of freshly prepared 10× NTP minus first strand buffer (0.5 M Tris, pH 8.0, 0.75 M KCl, 0.1 M DTT), 4 μl of 5 M betaine, and 1.2 μl of 100 mM MnCl_2_. Reactions were incubated at 25°C for 2 min before adding 1 μl of either SuperScript II RT enzyme or water (for no-RT controls). Reactions were carried out with the following program: 25°C for 10 min, 42°C for 90 min, 10 cycles of 50°C for 2 min followed by 42°C for 2 min, then 70°C for 10 min, and a hold at 12°C. Reactions were cleaned with G50 spin columns (Cytvia) prior to second strand synthesis. Second strand synthesis and library preparation were performed as described above for the 5NIA treatment.

### Chemical probing—in-cell DMS

HeLa cells were transfected in six-well plates with 1 μg of wild-type (WT) HIPK3 plasmid, as described above. At harvesting (48 h post-transfection), cells were washed with 1× phosphate buffered saline (PBS; Gibco) and allowed to equilibrate at 37°C in buffered media (DMEM, 200 mM Bicine, pH 8). Each well was treated with 100 μl of either 100% ethanol as a control or DMS diluted 1:20 in 100% ethanol at 37°C for 6 min. To quench the reaction, 1 ml of cold 20% BME was added to each well. After removing all liquid waste, RNA was isolated from cells with Trizol Reagent (Invitrogen) following the manufacturer’s protocol. To remove DNA, RNA was treated with TurboDNase (Invitrogen) following the manufacturer’s protocol, with the modification of treating for 1 h, adding 1 μl additional DNase halfway through. Following DNase digestion, RNA was purified by ethanol precipitation. For MaP-RT, 2 μg of RNA was used as template and reverse transcription was performed as described above for *in vitro* DMS probing. Reverse transcription reactions were cleaned up with G50 columns (Cytivia).

Library preparation for in-cell probing followed an amplicon-based strategy with four different primer sets (Supplementary Fig. S1A and Supplementary File S9). PCR-1 was performed with Q5 Hot Start High-Fidelity DNA Polymerase (NEB) using the entire 20 μl MaP-RT reaction and following the manufacturer’s protocol with 20 cycles of amplification. PCR-1 product was cleaned up with Ampure XP bead-based reagent (Beckman Coulter) following the manufacturer’s protocol. Product concentration was evaluated with Qubit dsDNA HS kit (Invitrogen) to determine input for indexing PCR (PCR-2) and rule out DNA contamination in no-RT controls. For PCR-2, 25 ng of input DNA was amplified using NEBNext Multiplex Oligos for Illumina (NEB) and NEBNext Ultra II Q5 Master Mix (NEB) according to the manufacturer’s protocol with half as much primer (0.25 μM final concentration) and 10 cycles of amplification. Products were cleaned up as in PCR-1, and purity verified with the Agilent 4200 TapeStation System. Libraries were pooled at equimolar concentrations and sequenced with the Illumina NextSeq 1000 platform.

### Structure probing data analyses

Structure probing data were primarily analyzed using Shapemapper2 v2.1.5 [27] and RNAvigate v1.0.0 [28], with structure prediction from RNAstructure version 6.4 [29]. For 5NIA data, the SHAPE profiles were rescaled according to [30] and values <−0.1 were set to −0.1 since any values less than zero have fewer mutations in the treated than the untreated control and are functionally zero. For DMS analyses, the input fasta file was modified to mask guanosine and uridine into lowercase and only analyze adenosine and cytidine nucleotides. SHAPE and DMS profiles, skyline plots, and correlation plots were generated with RNAvigate. Correlation coefficients were calculated with RNavigate. Minimum free energy structures were generated with Fold from the RNAstructure package. Pairing probabilities were generated with the partition function in RNAstructure followed by the ProbabilityPlot function. Maximum expected accuracy structures were generated using the MaxExpect function in RNAstructure. Structures were arranged for visibility and organization in StructureEditor 1.0, and then plotted with associated data and annotations in RNAvigate.

### Genome-wide analysis

CircRNA coordinates were collected from the circBase database [31]. Coordinates of all exons were collected from GENCODE v44. Alu element coordinates were identified from RepeatMasker annotations in Hg38 [32]. To categorize exons into circularizing and non-circularizing, annotations from circBase containing exactly one exon were intersected using BedTools Intersect [33] with annotations from GENCODE v44, with exons containing an overlap marked as circularizing.

Exon-flanking Alus were defined as full-length Alu elements within 2000 nucleotides of an exon. Therefore, exons were considered to have a 5′ flanking Alu if there was an Alu element that met the definition criteria on the 5′ side but not the 3′ side. Exons were considered to have a 3′ flanking Alu if there was an Alu element that met the definition criteria on the 3′ side but not the 5′ side. Exons were considered to have no Alu elements if no Alus meeting the definition criteria were found on either side. Exon-flanking Alus were considered inverted repeats if two Alus met the criteria on either side of the exon, and one Alu was found on the positive strand with the other Alu on the negative strand. Differences between groups were evaluated by taking the difference in the log odds ratio and compared statistically with Mann–Whitney *U* tests. To produce Alu element length distributions, downstream Alus were categorized as described above, regardless of length, into circularizing and non-circularizing and plotted based on length.

### Circularization assays

For circularization assays, constructs were transfected into six-well plates as described above. RNA was isolated using the Quick RNA Miniprep Kit (Zymo) with on-column DNase treatment, followed by an additional DNase treatment with TurboDNase (Invitrogen), and cleaned up again by ethanol precipitation. Preliminary circularization assays were performed with reverse transcriptase polymerase chain reaction (RT-PCR) and evaluated using gel electrophoresis.

RNA was subjected to quantitative reverse transcription polymerase chain reaction (qRT-PCR) analysis with custom TaqMan gene expression assays (Life Technologies). Reverse transcription was performed with SuperScript IV VILO Master Mix (Invitrogen) with ezDNase treatment using 500 ng RNA. Three different custom TaqMan probes (illustrated in Supplementary Fig. S8) were designed with the Life Technologies custom gene expression assay design tool corresponding to circRNA (assay ID: APFVV29), pre-mRNA (assay ID: APGZPM6), and lastly the neomycin resistance gene (assay ID: APEP2NU), which is specific to the plasmid and absent from the mammalian genome. The qPCR reactions were performed using the TaqMan Fast Advanced Master Mix for qPCR (Applied Biosystems) in 10 μl reactions [4.5 μl diluted complementary DNA (cDNA), 5 μl of 2× Master Mix, and 0.5 μl TaqMan probe]. For each qPCR reaction, 2 ng of cDNA per well was used for the circular and pre-mRNA probes, whereas 0.2 ng of cDNA per well was used for the neomycin probe. qPCR was performed on the Applied Biosystems QuantStudio 6 Flex Real-Time PCR System.

For data analysis, the neomycin resistance gene was used as a reference gene for qPCR to account for any variation in transfections. To quantify normalized circular and pre-mRNA, relative expression was calculated as 2^−ΔCt^, where ΔCt is the difference between the target and the reference (neomycin resistance). Circularization efficiency was calculated by taking the ratio of normalized circRNA to pre-mRNA and standard deviations were calculated with propagated error.

### SHAPE-JuMP probing

For SHAPE-JuMP probing, 1 μg of IVT RNA was denatured (65°C for 5 min and snap cooled on ice for 2 min) before being refolded in 2× Bicine folding buffer at 37°C for 15 min, followed by addition of 0.25 μg of 4′-aminomethyltrioxsalen hydrochloride per reaction (water for untreated) and incubation at 37°C for an additional 15 min. Samples were then cross-linked on ice with UV light at 365 nm for 10 min. Reactions were cleaned up with G50 columns (Cytiva).

For reverse transcription, 1 μl RT primer (JW39 or JW41, Supplementary File S9) was mixed with 9 μl of cross-linked RNA, denatured at 65°C for 5 min, followed by snap cooling on ice for 2 min. The RNA/primer mix was added to a reverse transcription reaction with 10 μl of 5× ThermoPol buffer made fresh (100 mM Tris–HCl, pH 8.8, 50 mM (NH_4_)_2_SO_4_, 50 mM KCl, 0.5% Triton X-100, 10 mM MgCl_2_), 1 μl of 10 mM dNTPs (NEB), 28 μl molecular biology grade water, and 1 μl C8 enzyme. Reverse transcription was performed at 65°C for 4 h as previously described [24]. Reverse transcription reactions were cleaned up with G50 columns (Cytiva).

Library preparation for JuMP experiments followed an amplicon-based strategy. PCR-1 (JW45&JW49 or JW48&JW50, Supplementary File S9) was performed with Q5 Hot Start High-Fidelity DNA Polymerase (NEB) following the manufacturer’s protocol for 25 cycles of PCR. PCR-1 reactions were cleaned up with Ampure XP bead-based reagent (Beckman Coulter) and concentrations were checked with Qubit dsDNA HS kit (Invitrogen). For PCR-2, 1 ng of PCR-1 product was used as template with TruSeq indexing primers (Illumina) and 10 cycles of PCR. PCR-2 product was cleaned up with Ampure XP bead-based reagent (Beckman Coulter). Library concentration and purity were verified using the Qubit dsDNA HS kit (Invitrogen) and the Bioanalyzer 2100 (Agilent), followed by equimolar pooling and sequencing on the Illumina MiSeq platform.

Sequencing data from SHAPE-JuMP experiments were analyzed with ShapeJumper V1.0 [23]. SHAPE-JuMP frequencies were calculated with using the data from normalizeDeletionRates.py in the ShapeJumper package, with low-count (<20) JuMP events omitted. Low-count JuMP events were omitted because they can be artificially high in this system due to both a low JuMP count and a low read depth (i.e. one event in one read is a 100% frequency), where the low read depth occurs on JuMPs deep into the read from the poor processivity of the reverse transcriptase. Values plotted are the differences in frequencies between the treated and untreated samples.

## Results

### Mutational profiling and structural analysis of inverted repeat pre-mRNA reveal Alu elements are likely structured

**Figure 1.**
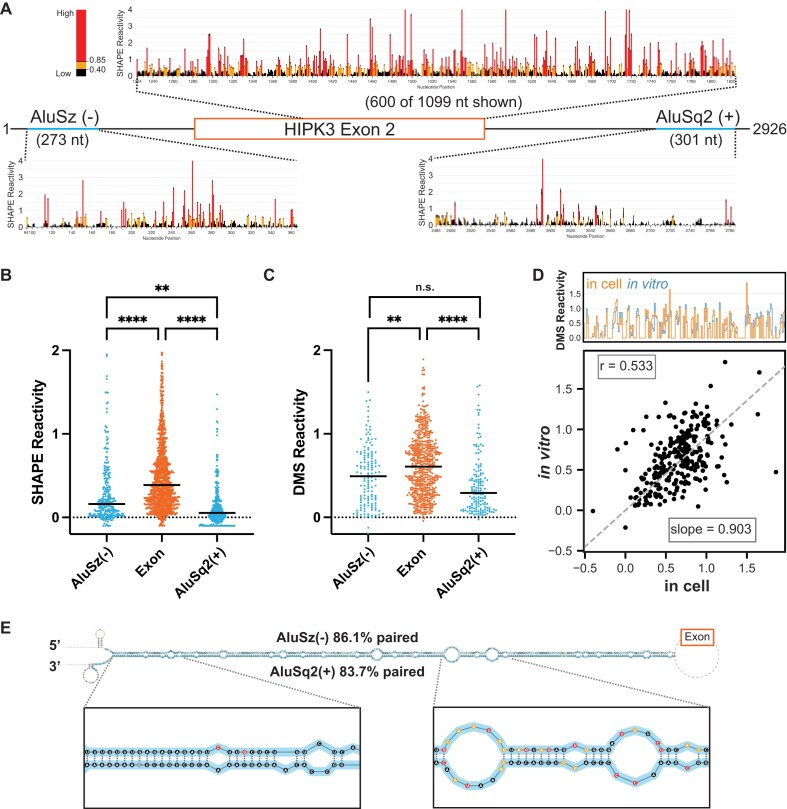
Chemical probing of *HIPK3* pre-mRNA. (**A**) Pre-mRNA gene diagram and SHAPE profiles of *HIPK3* exon 2 (orange) and its flanking Alu elements (blue). High SHAPE reactivities are shown in red, medium in orange, and low in black. (**B**) Median regional normalized SHAPE reactivities comparing each Alu (blue) and the exon (orange). Significance determined with an ordinary one-way ANOVA. Significance throughout all figures: *P < .05, **P < .01, ***P < .001, ****P < .0001. (**C**) Median regional normalized DMS reactivities comparing each Alu (blue) and the exon (orange). DMS reactivity analysis is limited to adenosine and cytidine nucleotides. Significance determined with an ordinary one-way ANOVA. (**D**) Comparison of normalized *in vitro* DMS reactivities with in-cell DMS reactivities. Top: skyline plot comparing per-nucleotide reactivity of a subset of the data. Bottom: linear regression and correlation comparison of normalized reactivities. Correlation calculated as Pearson’s correlation coefficient. (**E**) Maximum expected accuracy structure of the Alu–Alu hairpin informed by SHAPE probing. Structure is truncated for visualization and insets are zoomed in on representative regions. Alu elements are highlighted in blue and nucleotides are colored by SHAPE reactivity as in panel (A). Percentage paired reflects the percentage of Alu nucleotides that are paired to any other nucleotide.

We begin our investigation using SHAPE-MaP to chemically probe an *in vitro* construct of the human *HIPK3* pre-mRNA flanking exon 2. This construct is based on previous studies of the *HIPK3* circRNA, which has been shown to circularize via backsplicing and form a 1099-nucleotide circle [2, 15]. When transcribed, the total construct is a 2926-nucleotide RNA that includes a 273-nucleotide antisense AluSz(−) in the upstream intron and a sense 301-nucleotide AluSq2(+) in the downstream intron (Fig. 1A). Due to their inverted repeat configuration and high degree of complementarity, the two Alu elements are predicted to form base pairs across 85% of their sequence.

For SHAPE-MaP, we used the 5NIA to probe the RNA and performed nucleotide-specific normalization [30] to compare median SHAPE reactivities across different regions of the pre-mRNA independent of nucleotide content. We obtained two replicates of *in vitro* SHAPE-MaP experiments, analyzed the replicate correlation over several selected regions of the pre-mRNA (Supplementary Fig. S1A), and found that the replicates are highly correlated (Pearson’s correlation coefficient *r* = 0.83) (Supplementary Fig. S1B and C). Overall, the SHAPE profiles of the *HIPK3* Alu elements show a consistently lower reactivity than that of the exon region, implying that the Alu elements are more structured than the exon (Fig. 1A). Furthermore, the median SHAPE reactivities of both Alu elements are significantly lower than that of the exon (Fig. 1B). A similar trend is observed when considering the median adenosine and cytidine DMS reactivities for the same construct (Fig. 1C). The DMS data replicate with an *r* = 0.79 (Supplementary Fig. S1D and E).

In-cell pre-mRNA chemical structure probing data are more challenging to obtain, as introns are rapidly spliced and degraded. Using a tiled amplicon-based strategy, focusing on multiple amplicons targeting short regions, it is possible to collect chemical probing data at a limited scale [34]. We designed and tested multiple amplicons spanning the *HIPK3* pre-mRNA and identified four amplicons from which we were able to obtain reproducible in-cell DMS probing data (*r* = 0.7) (Supplementary Fig. S1A, F, and G). Previous work has shown that the in-cell environment creates more biological noise [35]; however, the overall pattern of the two replicates here is the same (Supplementary Fig. S1G).

Comparing the in-cell and *in vitro* DMS probing datasets (Supplementary Fig. S1H and particularly Supplementary Fig. S1I) reveals that reactivities in cells and *in vitro* are highly similar, suggesting that the *in vitro* data are representative of biologically relevant structures. Since the *in vitro* and in-cell probing data correlate well, we used the more comprehensive *in vitro* SHAPE-MaP data (Fig. 1A) to model the structure of the full-length circularizing pre-mRNA. This model contains a unique structure of the AluSz(−)/AluSq2(+) paired interaction (Fig. 1E), which is predicted to occur with high base-pairing probability (Supplementary Fig. S2A). Similar results are obtained when predicting the structure using DMS data (Supplementary Fig. S2B).

### Genomic and experimental measures of Alu-mediated long-range base pairing and circRNA formation

**Figure 2.**
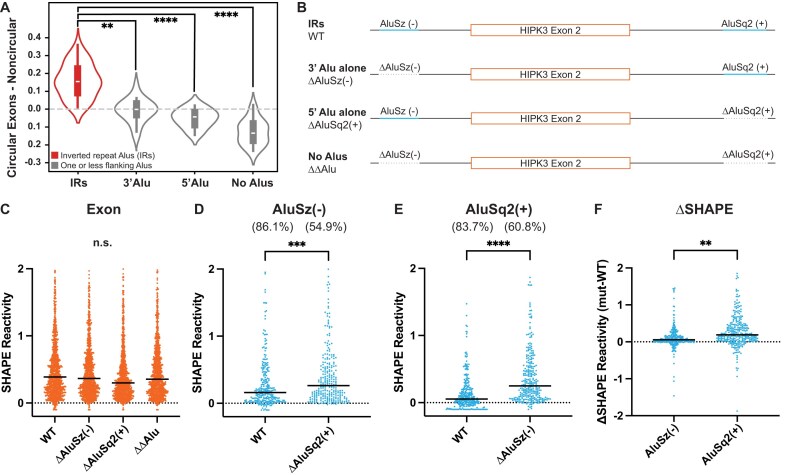
Inverted repeat Alu elements are required for circularization and likely base pair. (**A**) Violin plot showing enrichment for inverted repeat Alu elements in exons found in the circRNA database, circBase [31], compared to exons not reported to circularize, grouped by the pattern of Alu elements flanking the exon. IRs are inverted repeats (red), 3′ Alu lacks a 5′ Alu upstream of the exon, 5′ Alu lacks a 3′ Alu downstream of the exon, and No Alus have no Alu elements flanking the exon. *Y*-axis values are the difference in log odds ratios. Significance determined with Mann–Whitney *U* tests. Significance throughout all figures: *P < .05, **P < .01, ***P < .001, ****P < .0001. (**B**) Possible gene orientations for flanking Alu elements. Specifics for the *HIPK3* exon 2 system are shown. (**C**) Median SHAPE reactivity of *HIPK3* exon 2 across multiple Alu contexts. Significance determined with an ordinary one-way ANOVA test. (**D**) Median SHAPE reactivity of AluSz(−) with (WT) and without [ΔAluSq2(+)] its putative pairing partner. Numbers in parentheses are the percentages of AluSz(−) nucleotides paired in each context. Significance determined with a paired *t*-test. (**E**) Median SHAPE reactivity of AluSq2(+) with (WT) and without [ΔAluSz(−)] its putative pairing partner. Numbers in parentheses are the percentages of AluSq2(+) nucleotides paired in each context. Significance determined with a paired *t*-test. (**F**) Difference in SHAPE reactivities for each Alu, calculated by subtracting the SHAPE reactivity of each Alu in its putative pairing context (WT) from its SHAPE reactivity without its putative pairing partner (ΔAlu). Significance determined with an unpaired *t*-test.

We performed a genome-wide analysis of human circRNAs and measured a higher log odds likelihood of observing circularization if an exon is flanked by two inverted repeats compared to if an exon is flanked by a single or no Alu (Fig. 2A). The difference in log-likelihoods is small, albeit statistically significant (*P* < .01). Although it has been noted that over 80% of circular exons are flanked by intronic Alu elements [7], there are Alu elements flanking an almost equally large proportion of non-circularizing exons. As Alu elements can be classified into families and subfamilies based on sequence similarity, we performed an additional comparison of the log-likelihood of observing circularization based on Alu family and subfamily and orientation (Supplementary Fig. S3). Notably, there are only small differences in log-likelihood differences between families. Thus, although circularization seems to be slightly more likely if inverted flanking Alus are present, inverted flanking Alu elements alone are not a strong predictor as to whether a circRNA will form.

Since the presence of flanking Alu elements does not guarantee backsplicing, we next focused on constructs that deleted one or both of the Alu elements flanking *HIPK3* exon 2 (Fig. 2B) to experimentally investigate corresponding 3′, 5′, and No Alu, both structurally and functionally. We initially compared the median SHAPE reactivity for the 1099-nucleotide exon 2 for the WT and three constructs that deleted either 5′, 3′, or both Alu elements (Fig. 2C). Overall, the median SHAPE reactivity in the exon is not significantly different across all constructs (Fig. 2C). In contrast, the median SHAPE reactivity of each Alu element is significantly higher if the opposite Alu is absent, as compared to the WT context (Fig. 2D and E). The increase in reactivity without its pairing partner is more dramatic for AluSq2(+) than it is for AluSz(−) (Fig. 2F). Similar trends are also observed in the DMS data (Supplementary Fig. S4), suggesting that each Alu is less paired when its partner is absent from the same pre-mRNA. Furthermore, when observed on a per-nucleotide basis (Supplementary Fig. S5) the difference in reactivity, as measured by ΔSHAPE [26, 36], is primarily driven by an overall change across the entire Alu element, although a few local regions do exhibit larger changes.

When using the SHAPE data to model the secondary structures of the ΔAluSq2(+) (Supplementary Fig. S6) and ΔAluSz(−) (Supplementary Fig. S7) deletion constructs, inter-intron pairs are no longer predicted as expected. Instead, the remaining Alu element in each deletion construct is predicted to form interactions between the Alu element and its flanking intronic sequences (Supplementary Figs S6 and S7A–C). Folding the AluSq2(+) sequence in isolation combined with the ΔAluSz(−) SHAPE data provides a more traditional Alu-like fold (Supplementary Fig. 7D and E). Overall, the structural data and reactivity changes in the deletion mutants indirectly suggest that long-range base-pairing interactions are occurring in our WT pre-mRNA construct and that deletion of either Alu (or both) eliminates inter-intron base pairing.

### Functional characterization of all constructs and partial deletions of AluSq2(+)

**Figure 3.**
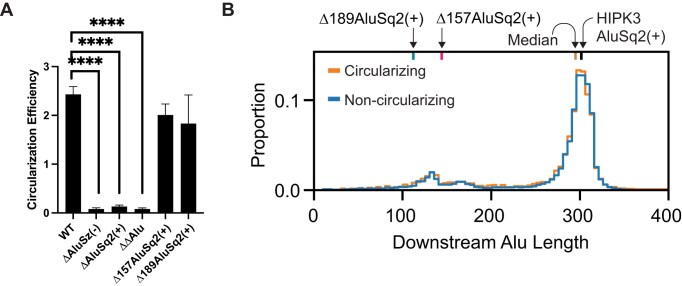
Alu elements are required and partial Alu elements are sufficient for circularization. (**A**) Circularization efficiency of *HIPK3* exon 2 in differing Alu contexts. Significance determined with an ordinary one-way ANOVA and Dunnett’s multiple comparisons test. Significance throughout all figures: *P < .05, **P < .01, ***P < .001, ****P < .0001. (**B**) Distribution of the length of Alu elements downstream of exons. Alu elements associated with exons in the circRNA database, circBase, are shown in orange, whereas Alu elements associated with exons not known to circularize are in blue.

To examine the effects of the potential pairing interaction on backsplicing, we designed a qRT-PCR assay to measure the circularization efficiency of different constructs (Supplementary Fig. S8A). We first performed RT-PCR assays to confirm our ability to detect HIPK3 circRNA and pre-mRNA with specificity (Supplementary Fig. S8B). None of the deletion mutants—ΔAluSz(−), ΔAluSq2(+), or ΔΔAlu—produce circles (Fig. 3A), emphasizing that circularization is dependent on the presence of the both AluSz(−) and AluSq2(+), consistent with previous studies [2]. To identify the length of the Alu–Alu base-pairing interaction required for circularization, we designed partial deletions of AluSq2(+), where 157 or 189 nucleotides are deleted from the 5′ end of AluSq2(+). The partial deletion constructs, Δ157AluSq2(+) and Δ189AluSq2(+), circularize with similar efficiency as WT (Fig. 3A). These results suggest that circularization is dependent on inter-intron base pairing but that partial length inverted repeats are sufficient.

To further examine alternative pairing possibilities, we generated and tested circularization in two additional constructs where we manipulated the Alu configuration in terms of location and orientation (Supplementary Fig. S9A). First, we positionally swapped AluSz(−) and AluSq2(+), such that AluSq2(+) was the upstream Alu and AluSz(−) was now the downstream Alu. The position of the Alus had no effect on circularization (Supplementary Fig. S9B), and the relative SHAPE reactivity of the Alu elements was specific to each Alu element and not their position (Supplementary Fig. S9C). Next, we created a reverse complement version of both Alu elements, which retains sequence complementarity across the two Alu elements, but changes AluSz(−) to AluSz(+) and AluSq2(+) to AluSq2(−) (Supplementary Fig. S9A). The reverse complement construct was also able to circularize, even more efficiently than WT (Supplementary Fig. S9B), and again demonstrated SHAPE reactivity specific to each Alu element independent of the orientation of the Alu element (Supplementary Fig. S9D). However, the increased circularization efficiency of the reverse complement construct may be due to a loss of pre-mRNA rather than an increase in circRNA (Supplementary Fig. S8B). Notably, both the swapped and reverse complement constructs demonstrate decreased SHAPE reactivity in the Alu elements, suggestive of Alu–Alu base pairing. Together, these results suggest that changing the orientation or position of the Alus does not negatively affect backsplicing.

To further identify whether circularization requires a specific length of Alu–Alu pairing interaction, we performed a genome-wide analysis of human circularizing and non-circularizing exons flanked by inverted Alu repeats and plotted the distribution of the length of the downstream Alu (Fig. 3B). We observed no difference in the distributions, consistent with our HIPK3 circularization assay and our partial AluSq2(+) deletion mutants. Together, these results suggest that ∼100 nucleotides of complementary sequence are sufficient to favor circularization, and that two full-length Alu repeats are not essential.

### Direct probing of long-range interactions using SHAPE-JuMP

The structural data presented thus far are consistent with a model in which the HIPK3 exon 2 flanking AluSz(−) and AluSq2(+) form an extended secondary structure. To directly measure the Alu–Alu interaction, we used SHAPE-JuMP [23, 24] to obtain nucleotide resolution information on long-range interactions in the WT, Δ157AluSq2(+), Δ189AluSq2(+), ΔAluSz(−), and ΔAluSq2(+) constructs. In SHAPE-JuMP experiments, a chemical cross-linker (here, a psoralen cross-linker) covalently links nucleotides that are in close three-dimensional proximity. The cross-links are then “read” using a highly processive reverse transcriptase that “jumps” across the cross-links, recording the cross-linked interaction as a deletion. The resulting cDNA is sequenced and deletion rates for specific nucleotide pairs are counted by massively parallel sequencing [23, 24]. We used an amplicon-based strategy to capture long-range interactions using short-read sequencing and to specifically capture inter-intron jumps.

**Figure 4.**
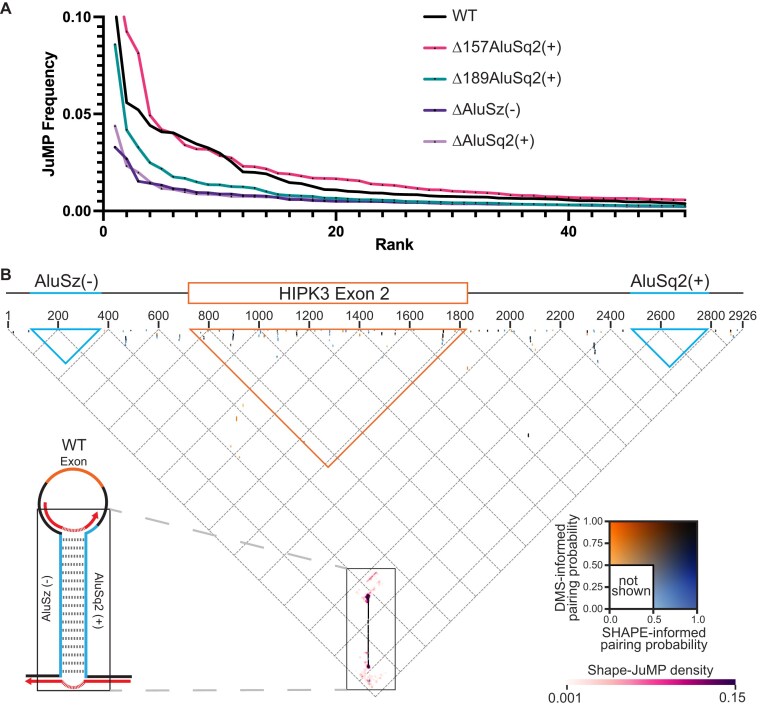
JuMP, SHAPE, and DMS data are in agreement. (**A**) JuMP event frequency distribution, where the JuMP frequency is plotted on the *y*-axis sorted from highest to lowest frequency (rank, *x*-axis). The amount of high-frequency JuMPs corresponds to the amount of predicted Alu pairing. (**B**) The inverted triangle represents all possible pairings for the WT *HIPK3* construct, where the horizontal axis is the 5′ to 3′ sequence, as illustrated by the gene diagram (top). The blue triangles represent the space occupied by each Alu element, whereas the orange represents the exon. Dots on the triangle represent predicted base-pairing interactions, colored by pairing probability informed by each probe (DMS or SHAPE) as seen in the inset color key on the right. Interactions based on JuMP data are represented by heatmaps (pink clouds) and shown as JuMP density.

We first evaluated the cumulative ranked distribution of observed reverse transcriptase jump frequency (deletions normalized to read depth) for the five probed constructs (Fig. 4A). The highest jump frequency is observed for the WT and Δ157AluSq2(+). The Δ189AluSq2(+) construct has an intermediate level of jumps, whereas both ΔAluSq2(+) and ΔAluSz(−) have very few jumps that are comparable to background noise. The jump frequency for each construct is consistent with its circularization efficiency, suggesting that the structures detected by -JuMP are important for circularization. We performed -JuMP experiments with circularizing constructs in replicate (Supplementary Fig. S10); in subsequent analyses, both replicates have been combined into a single dataset.

**Figure 5.**
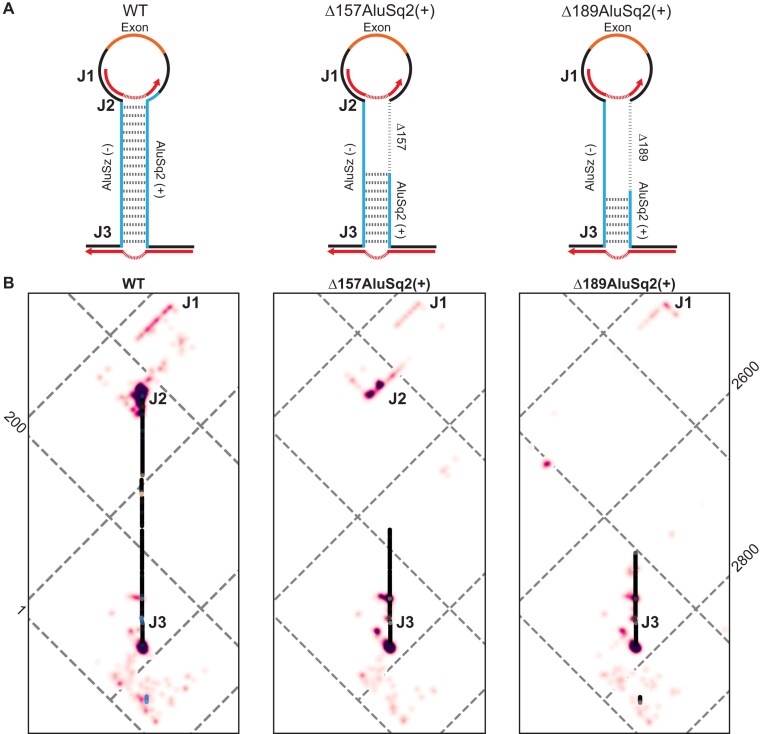
JuMP data of circularizing constructs mapped to WT coordinates show broad structural agreement. (**A**) Model structure illustrations for WT and partial Alu deletions, Δ157AluSq2(+) and Δ189AluSq2(+). Alu elements are illustrated in blue with deleted sequence marked by a dashed line and the exon in orange. Red arrows represent amplicon-targeted predicted JuMP events, with the deletion marked by the dashed portion. Approximate locations of notable JuMP events are denoted by J1, J2, and J3. (**B**) Zooming in on the Alu–Alu interaction of WT, Δ157AluSq2(+), and Δ189 AluSq2(+) structures, mapped to WT coordinates. JuMP density is colored as in Fig. 4B, as is pairing probability for WT. Pairing probabilities for Δ157AluSq2(+) and Δ189AluSq2(+) are grayscale colored based only on SHAPE-informed pairing probabilities. Notable JuMP interactions are denoted by J1, J2, and J3.

We use a “triangular” representation to visualize the -JuMP data in the context of the secondary structure model (Fig. 4B) [37]. Several important features of the SHAPE and DMS informed structural models for the WT construct are clear when superimposed and colored by pairing probability (Fig. 4B). Most of the structures observed (dots representing base pairs) are short-range local structures, as indicated by their positions near the top of the graph. Among these small local structures, there is some variance between structures that are SHAPE-supported, DMS-supported, or both. However, almost all the dots representing the AluSz(−)/AluSq2(+) interaction are black (near the bottom point of the triangle diagram), confirming that the SHAPE and DMS informed base-pairing probabilities are both in agreement and very high (∼1.0) (Fig. 4B, enlarged in Fig. 5). We observe no alternative base paring in either the SHAPE or DMS informed structures in proximity to the AluSz(−)/AluSq2(+) pairs (Fig. 4B). Importantly, there are no long-range base-pairing interactions connecting the splice sites or near where the ends of the exon would come together for backsplicing (the tip of the orange triangle), suggesting that base pairing near the backsplice junction is not occurring; instead, the only predicted long-range base pairing is the AluSz(−)/AluSq2(+) interaction.

Therefore, the -JuMP experiments were designed to target the predicted AluSz(−)/AluSq2(+) interaction in the WT construct. The most prominent jumps are observed at both ends of the AluSz(−)/AluSq2(+) hairpin (labeled as J2 and J3) in the WT construct (Figs 4B and 5). These experiments exhibit very few inter-Alu cross-link jumps inside the hairpin; most jumps are concentrated in non-paired regions (J1) and at the very ends of the paired region (J2 and J3) (Fig. 4B). These observations are consistent with the ability of SHAPE-JuMP to detect both secondary structures and tertiary (non-base-pairing) interactions [23, 24]. The detection of jumps at both ends of the AluSz(−)/AluSq2(+) hairpin provides direct evidence for the existence of this long-range interaction.

**Figure 6.**
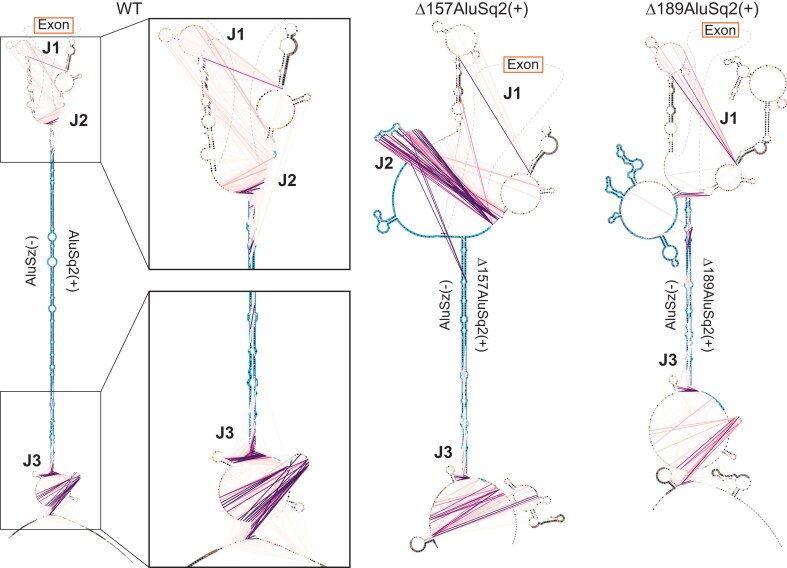
Maximum expected accuracy secondary structure diagrams of the Alu–Alu interaction of WT, Δ157AluSq2(+), and Δ189AluSq2(+) constructs. Structures are informed by SHAPE chemical probing. Alu elements are highlighted in blue, with primer locations from JuMP highlighted in dark gray. JuMP interactions are shown by pink/purple lines, with the color scale from Fig. 4B. Individual nucleotides are colored by SHAPE reactivity. Structures are truncated to focus on Alu elements and JuMP interactions. Notable JuMP interactions are denoted by J1, J2, and J3 and correspond to the same labels as in Fig. 5.

When comparing the three constructs that circularize [WT, Δ157AluSq2(+), and Δ189AluSq2(+)] by mapping the deletion mutants to WT coordinates, there is a consistent pattern of jumps that directly detect and further support the existence of the Alu–Alu hairpin (Fig. 5). All three structures show nearly identical JuMP interactions that directly indicate Alu–Alu pairing (J3) (Fig. 5). The construct with the shortest Alu–Alu interaction [Δ189AluSq2(+)] shows the most internal Alu–Alu hairpin jumps (Figs 5 and 6), suggesting that the lack of inter-Alu jumps deep in the Alu–Alu hairpin in the WT construct is likely due to the extremely high level of cross-linking and significant base pairing of two full-length inverted Alu elements, as these factors limit the processivity of the reverse transcriptase resulting in fewer sequencing reads deep into the hairpin.

The patterns of jump density for the WT, Δ157AluSq2(+), and Δ189AluSq2(+) are broadly similar for non-inter-Alu pairing (Fig. 5). Specifically, there is a very similar pattern of density at the bottom of the AluSz(−)/AluSq2(+) for all three constructs (J3) extending beyond the hairpin and representing the areas nearest the 5′ and 3′ ends of the constructs (Fig. 5). All three also show a similar pattern of jumps on the exon-proximal side of the Alu elements (J1) (Fig. 5). These regions lack sequence complementarity and are not predicted to be paired in any of the structure models, suggesting the cross-link jumps are reporting three-dimensional spatial proximity in these regions.

The same data can also be visualized on more traditional secondary structure diagram models of each circularizing construct [WT, Δ157AluSq2(+), and Δ189AluSq2(+)] (Fig. 6). These secondary structure diagrams reveal the extent to which many of the observed jumps are not between canonically base-paired regions. Interestingly, despite the removal of 157 nucleotides in AluSq2(+), the J2 interaction at the exon-proximal end of the Alu elements is maintained across WT and Δ157AluSq2(+). The removal of an additional 32 nucleotides in Δ189AluSq2(+) abolishes this J2 interaction; however, -JuMP still detects the base pairing at both ends of the truncated AluSq2(+) in Δ189AluSq2(+) (Fig. 6). In effect, the AluSz(−)/AluSq2(+) hairpin brings into close three-dimensional proximity two intronic regions (J1 and J3) causing them to interact spatially. These interactions are preserved across all three circularizing constructs [WT, Δ157AluSq2(+), and Δ189AluSq2(+)]. Thus, our data suggest that the ability of inverted repeats to lock two introns together is spatially orienting the 5′ and 3′ splice sites, which facilitates backsplicing.

## Discussion

Inverted repeats like the AluSz(−)/AluSq2(+) interaction in *HIPK3* exon 2 appear to form highly stable, fully paired interactions, even though they are over 2000 nucleotides apart. In our study, these long-range interactions are directly supported by extensive chemical probing data. These extended pairing interactions, in turn, create sufficient proximity between intronic structured elements that they are detectable by chemical cross-linking and SHAPE-JuMP. This spatial proximity is sufficient to facilitate backsplicing. This work represents the first direct experimental detection and structural description of a long-range Alu–Alu interaction at single-nucleotide resolution.

As implemented in this study, SHAPE-JuMP utilizes gene-specific reverse transcription followed by a targeted amplicon-sequencing approach, which limits detection to sites that are suspected to interact *a priori*. Here, we used our knowledge of the well-studied HIPK3 system to design a priming strategy that targeted these regions and produced amplicon products that are of an appropriate size for short-read Illumina sequencing. This is a key step because SHAPE-JuMP creates a deletion where the RNA is cross-linked, which affects the size of the amplicon. Future development of the SHAPE-JuMP approach could support in-cell cross-linking followed by -JuMP reverse transcription, opening the door for improved sequencing strategies that would allow for transcriptome-wide analysis of long-range through-space RNA structure. Such development would enable the evaluation of the potential regulatory roles of Alu–Alu interactions in determining splicing outcomes, including alternative splicing and backsplicing. Targeted priming in Alu-rich regions in pre-mRNA is still a significant challenge, however, due to the sequence complexity of and around Alu repeats, coupled with the low cellular abundance of pre-mRNA.

Previous studies using reporter systems analogous to the one used here have found that overexpression can generate various unexpected trans-spliced RNAs [38]. In our splicing assays, we do not observe off-target splicing (Supplementary Fig. S8B). Furthermore, our SHAPE-JuMP experiments were performed on IVT RNAs that were verified to be a single product by gel electrophoresis. The very low abundance of pre-mRNAs in cells would not allow us to perform these experiments on endogenous systems. We cannot exclude that some of our findings will not precisely recapitulate endogenous structures. However, given the high thermodynamic stability of inverted repeat Alu–Alu interactions, it is likely that the structures measured here are present in the endogenous pre-mRNA.

Splicing and backsplicing are governed by the same splicing code, which primarily consists of short sequence motifs. As in linear splicing, backsplicing requires the essential splice site sequence motifs [39]. Notably, backsplicing can be extensively regulated by splicing regulatory elements (SREs), as is linear splicing; however, the effects of the same SREs on backsplicing are variable and sometimes even opposite that of linear splicing [39]. RNA secondary structure also contributes to splicing [1, 34, 40]. Indeed, a combination of both key sequence motifs and local RNA structure is a stronger prediction of alternative splicing outcomes than either factor alone [34]. Here, we describe long-range tertiary structural interactions and extensive Alu–Alu base pairing that could potentially increase the initial efficiency of spliceosome formation around the initial exon definition step and thereby facilitate backsplicing [41]. Our findings are consistent with a complex, three-dimensional RNA structure scaffold that can influence spliceosome assembly [42–44]. Overall, our results emphasize that the Alu–Alu interactions notably enhance backsplicing, but do not directly speak to the effects of other splicing factors. Future work could further examine how the structural modulation of the pre-mRNA can influence outcomes of linear versus backsplicing.

In this work, we experimentally explored the pre-mRNA structures that facilitate backsplicing in the *HIPK3* exon 2 circRNA. Minimized Alu elements of the HIPK3 system as short as 31 nucleotides for AluSz(−) and 141 nucleotides for AluSq2(+) are sufficient for circularization [2]. Here, we explored the RNA structure in the WT Alu context of the HIPK3 pre-mRNA, as well as truncations of AluSq2(+) modeled after previous studies [2, 16]; however, instead of minimizing both the Alu elements, we maintained the native upstream AluSz(−) to better mimic the natural system. Truncating AluSq2(+) to its minimized 141-nucleotide length [Δ157AluSq2(+)] and beyond [109 nucleotides in Δ189AluSq2(+)] still permitted circularization when paired with its full-length AluSz(−) partner and maintained both Alu–Alu interactions and complex tertiary interactions beyond the Alu elements. We coupled this experimental work with genome-wide analyses that further support a complex, and likely structural, explanation of backsplicing beyond inverted sequence complementarity. Genomic analyses suggested that the presence of exon-flanking inverted repeat Alu elements is only modestly predictive of exon circularization, as the log-likelihood of circularization is only marginally larger for exons flanked by inverted repeats versus not (Fig. 2A). Furthermore, the length of the predicted Alu–Alu interaction is insufficient to identify those exons capable of circularizing (Fig. 3B). Combined with the structural data presented here, the presence and length of inverted repeat Alu elements are not strong predictive features of circularization alone. Our experimental data reveal a more complex induced proximity that is conserved in all circularizing constructs.

Although this work focuses on RNA structure as a contributing factor to backsplicing, there are other factors that could contribute or interact with RNA structure and remain unexplored. For example, inverted repeat Alu elements around circularizing exons that are predicted to pair have been shown to have higher frequencies of ADAR editing; however, knocking out ADAR1 and ADAR2 proteins has been shown to increase backsplicing, suggesting that ADAR editing functions to limit backsplicing [7]. Furthermore, DHX9 specifically binds to inverted repeat Alu elements and represses backsplicing [17]. ADAR editing and DHX9 act to recognize long double-stranded RNA structure, features we did not examine in our experimental system.

The structures of inverted repeats present a unique challenge in structure prediction. Inverted repeat Alu elements are highly complementary and are predicted by traditional thermodynamic modeling to form large hairpins even when separated by long stretches of sequence. In the WT *HIPK3* construct, the AluSz(−)/AluSq2(+) interaction occurs in the minimum free energy structure and has 100% base-pairing probability in the Boltzmann suboptimal ensemble. The high degree of complementarity drives an enormous thermodynamic favorability that disfavors the possibility of alternative (plausible) structures, even if there are large distances between inverted repeats.

Alu elements comprise ∼10% of the human genome [8]; nonetheless, the abundance of repetitive sequences in the human genome has only recently been characterized fully, as part of the telomere to telomere genome [45]. Given that a majority of the genome is also transcribed, the potential for inverted repeat base pairing in the transcriptome is enormous. These inverted repeat interactions have the potential to bring diverse RNA sequences, nominally kilobases apart in primary sequence, into physical proximity. Determining the specificity with which two inverted repeats form a long-range interaction is challenging for repetitive sequences. In our experiments, we leveraged the unique ability of the JuMP-RT, coupled with specific priming outside of the repetitive sequences, to directly measure this interaction. Our experiments also revealed persistent non-base-pairing interactions outside complementary Alu sequence regions. These data suggest that noncanonical through-space interactions in a pre-mRNA may be common and imply that secondary structure considerations alone are insufficient to understand, or ultimately predict, specific aspects of splicing regulation.

## Supplementary Material

gkaf433_Supplemental_Files

## Data Availability

Sequencing data are accessible through Gene Expression Omnibus (GEO) accession number GSE283716. DMS and SHAPE reactivities calculated for all the constructs are available as supplementary files through GEO. SRA data for all the constructs (BioProject ID: PRJNA1195085) can be accessed from NCBI using the following link: https://www.ncbi.nlm.nih.gov/geo/query/acc.cgi?acc=GSE283716.

## Supplementary data

Supplementary data is available at NAR online.

## Notes

Present address: Molecular Biophysics and Biochemistry, Yale University, New Haven, CT 06520, United States
