# Supplementary material for: Structural determinants of inverted Alu-mediated backsplicing revealed by -MaP and -JuMP
Source: Nucleic Acids Res. 2025 May 21;53(9):gkaf433. doi: 10.1093/nar/gkaf433 (PMC12093144; doi:10.1093/nar/gkaf433)
Supplement: gkaf433_Supplemental_Files [file gkaf433_supplemental_files.zip › Supplementary_figures_and_captions_for_upload.pdf]

Structural determinants of inverted Alu-mediated backsplicing revealed by -MaP and -JuMP

Justin M Waldern[1], Colin Taylor[1], Catherine A. Giannetti[2], Patrick S. Irving[2], Scott R. Allen[1], Mingyi Zhu[3,b] Rolf Backofen[4,5], Dave Mathews[3], Kevin M. Weeks[2] and Alain Laederach[1,a]

Supplementary Figures and Captions

Supplementary Figure 1: Replicate comparisons

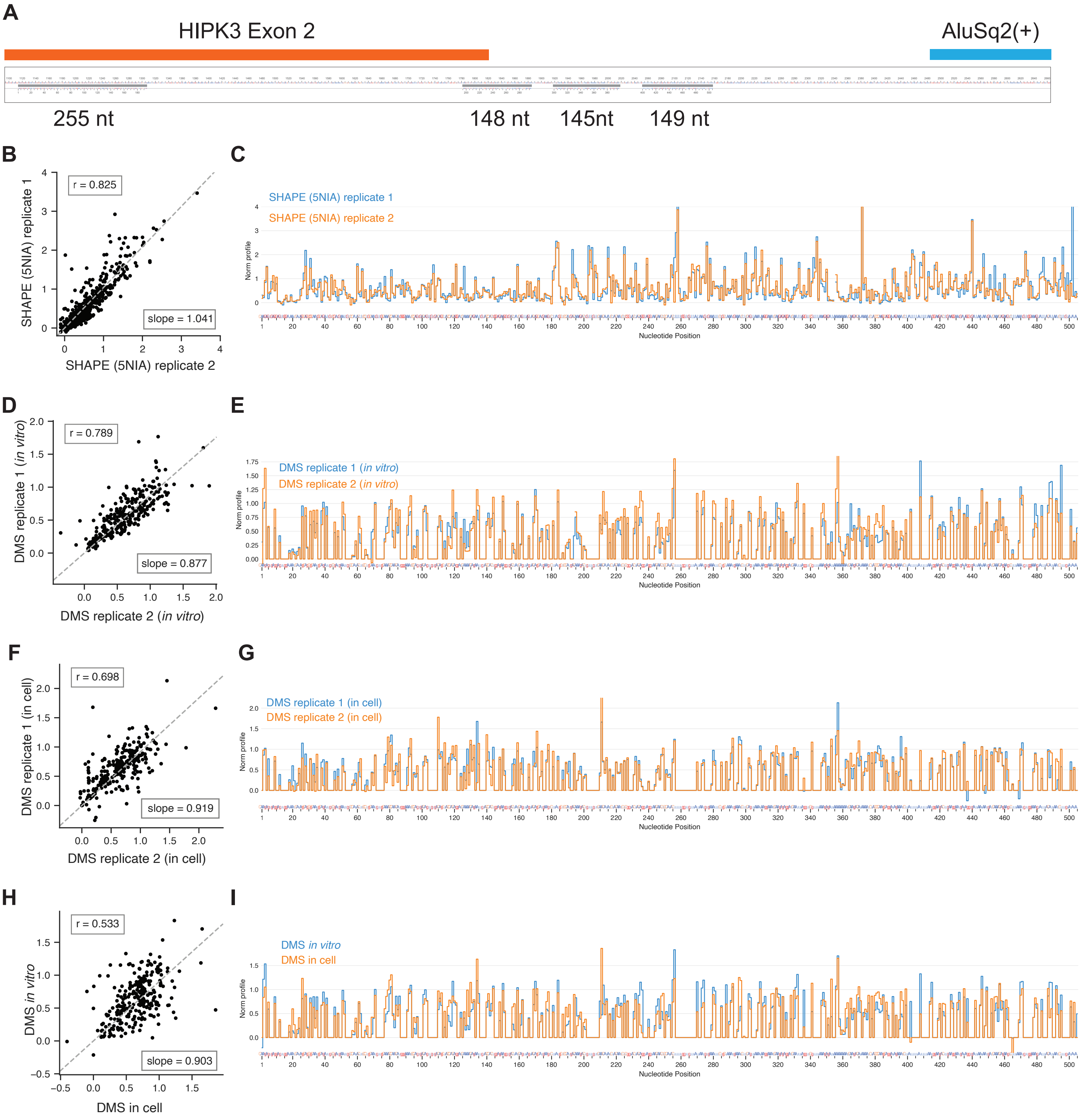

Supplementary Figure 1: Chemical probing replicates correlate. **A.** Gene diagram of how in cell amplicons (bottom, gray) align to the entire *in vitro* construct (top). Exon (orange) and AluSq2(+) (blue) features are colored. Aligned regions (grey, middle) are used to evaluate correlations throughout this figure to allow for comparison between all data types. **B.** Linear regression of two replicates of *in vitro* SHAPE (5NIA) probing of the wild-type construct. Values are normalized SHAPE reactivity. **C.** Skyline plot of normalized SHAPE profiles of two replicates of *in vitro* SHAPE (5NIA) data. Values are normalized SHAPE reactivity. **D.** Linear regression of two replicates of *in vitro* DMS probing of the wild-type construct. Values are normalized reactivities of only adenosine and cytidine nucleotides. **E.** Skyline plot of normalized reactivity of two replicates of *in vitro* DMS probing. Values are normalized reactivities, however guanosine and uridine nucleotides were not analyzed and set to 0 for both datasets. **F.** Linear regression of two replicates of in cell DMS probing of the wild-type construct. Values are normalized reactivities of only adenosine and cytidine nucleotides. **G.** Skyline comparison of two replicates of in cell DMS probing. Values are normalized reactivities, however guanosine and uridine nucleotides were not analyzed and set to 0 for both datasets. **H.** Linear regression comparing in cell DMS probing with *in vitro* DMS probing. Values are normalized reactivities of only adenosine and cytidine nucleotides. **I.** Skyline comparison of in cell DMS probing against *in vitro* DMS probing. Values are normalized reactivities, however guanosine and uridine nucleotides were not analyzed and set to 0 for both datasets.

Supplementary Figure 2: Wild-type structures

A SHAPE (5NIA) informed arcplots

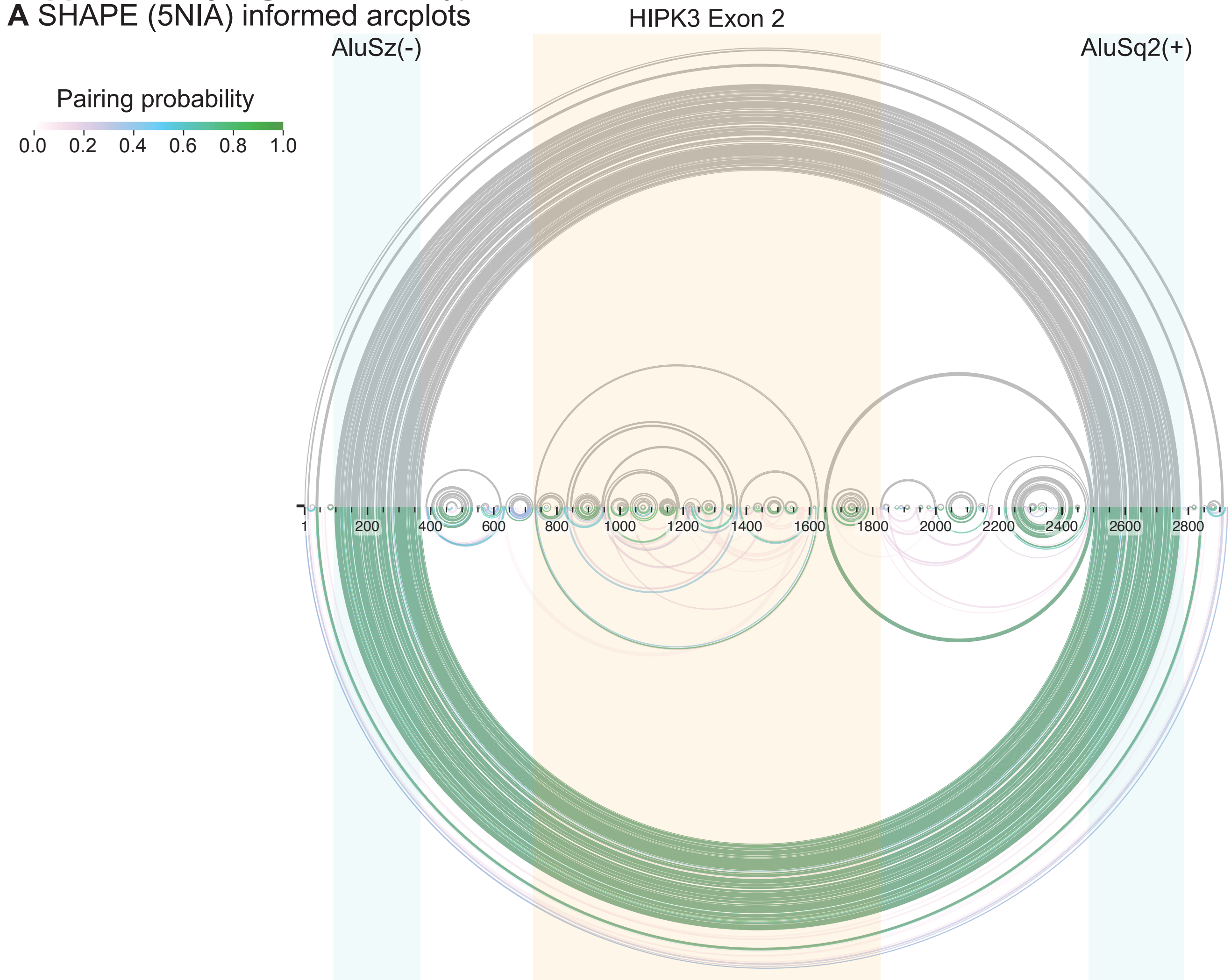

B DMS-informed arcplots

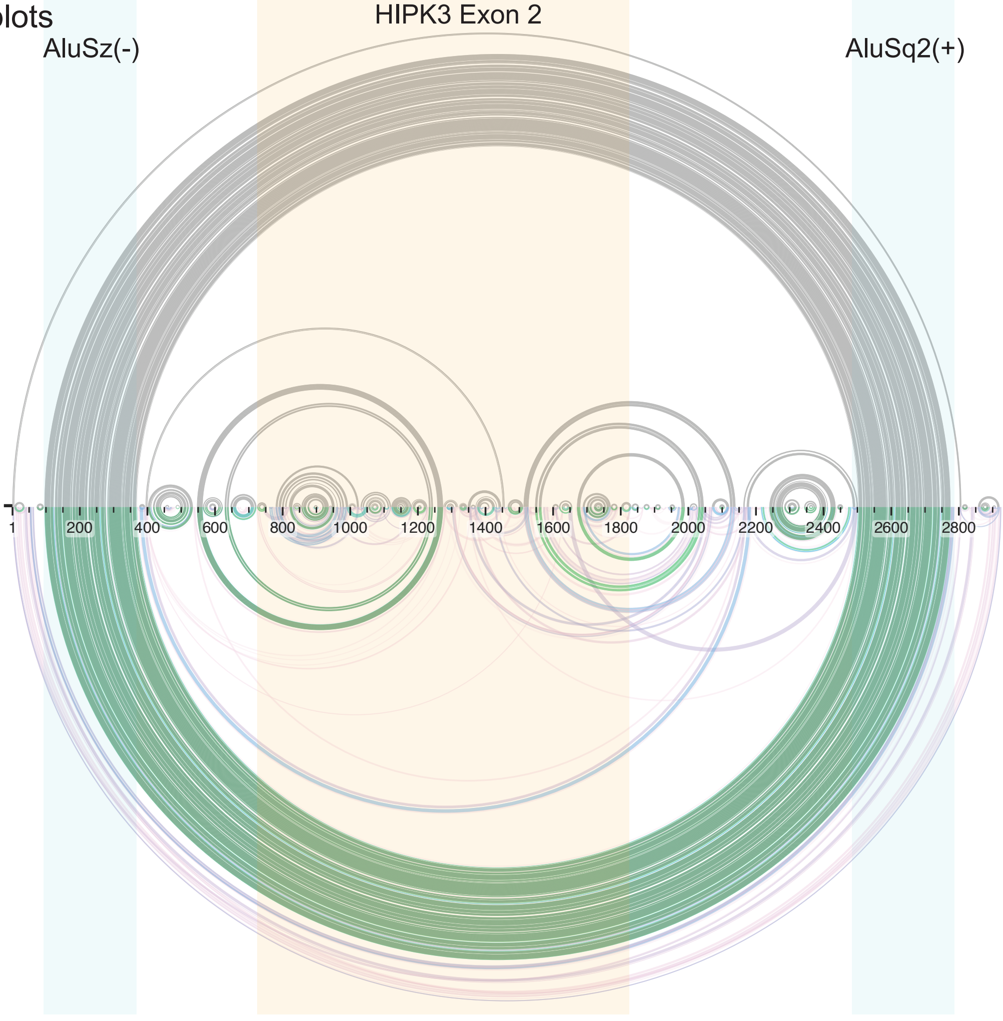

Supplementary Figure 2: Wild-type secondary structures (arcplots) **A.** SHAPE-informed arcplots. The blue shaded regions indicate the Alu elements, whereas the orange shaded region is the exon. The top arcs represent the minimum free energy structure, whereas the bottom represent pairing probabilities. **B.** DMS-informed arcplots. The blue shaded regions indicate the Alu elements, whereas the orange shaded region is the exon. The top arcs represent the minimum free energy structure, whereas the bottom represent pairing probabilities.

Supplementary Figure 3: Difference in log odds ratio between circRNA and non-circularizing exons, comparing Alu family and orientation.

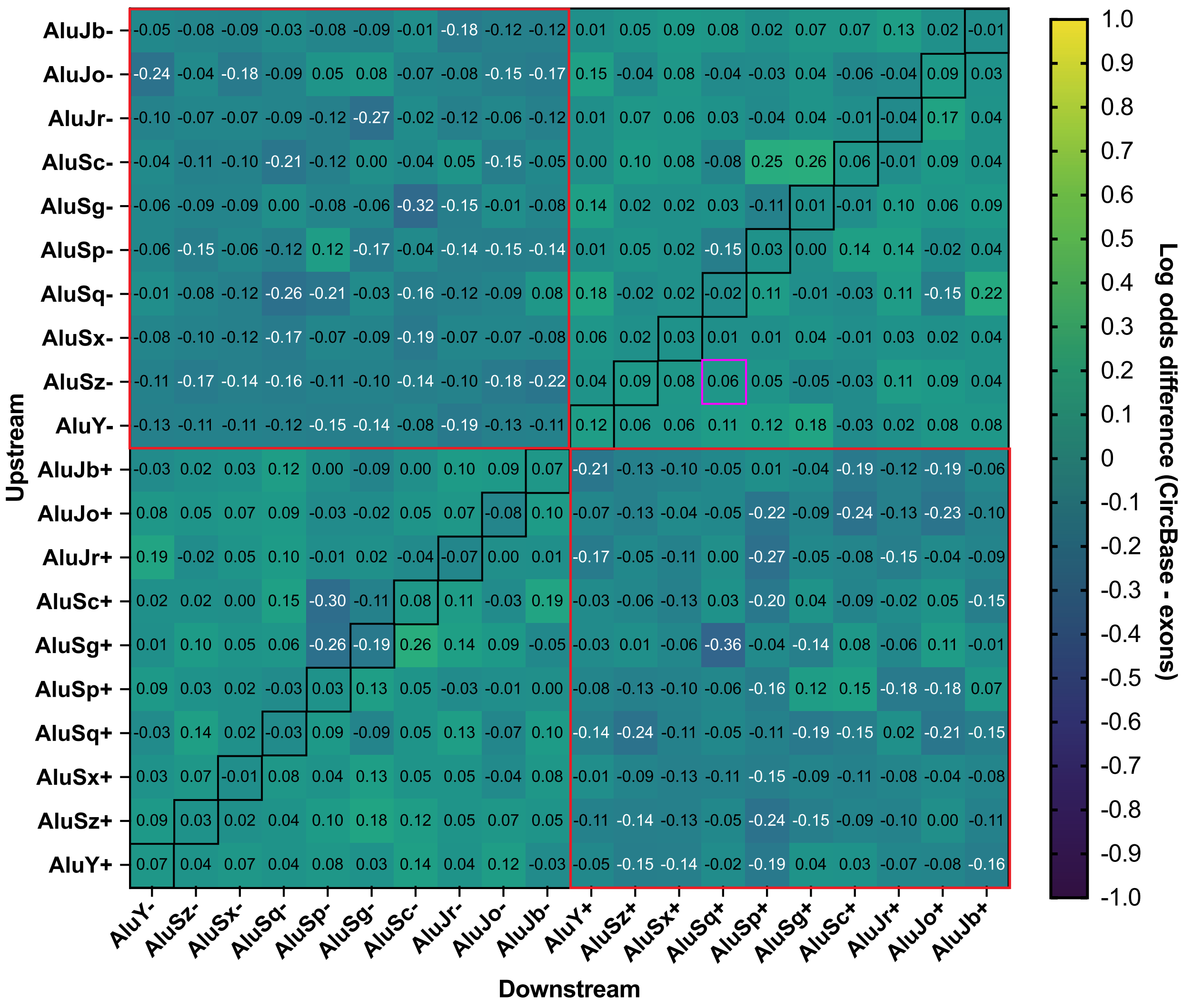

Supplementary Figure 3: Difference in log-odds ratio between circRNA and non-circularizing exons, comparing Alu family and orientation. The x-axis represents downstream Alu elements (Alus following the exon), whereas y-axis represents the upstream pairing partner. Large red boxes illustrate comparisons between same-orientation Alu elements (i.e., direct repeats). Small black boxes highlight the comparison between same-family Alu elements that are inverted repeats (e.g., AluY+ with AluY-). A magenta box highlights the AluSz-/AluSq+ pairing that is inclusive of the experimental HIPK3 system used here. Heatmap coloring represents the difference in log likelihoods compared to expected probability of circularization.

Supplementary Figure 4: DMS Regional Median Reactivity

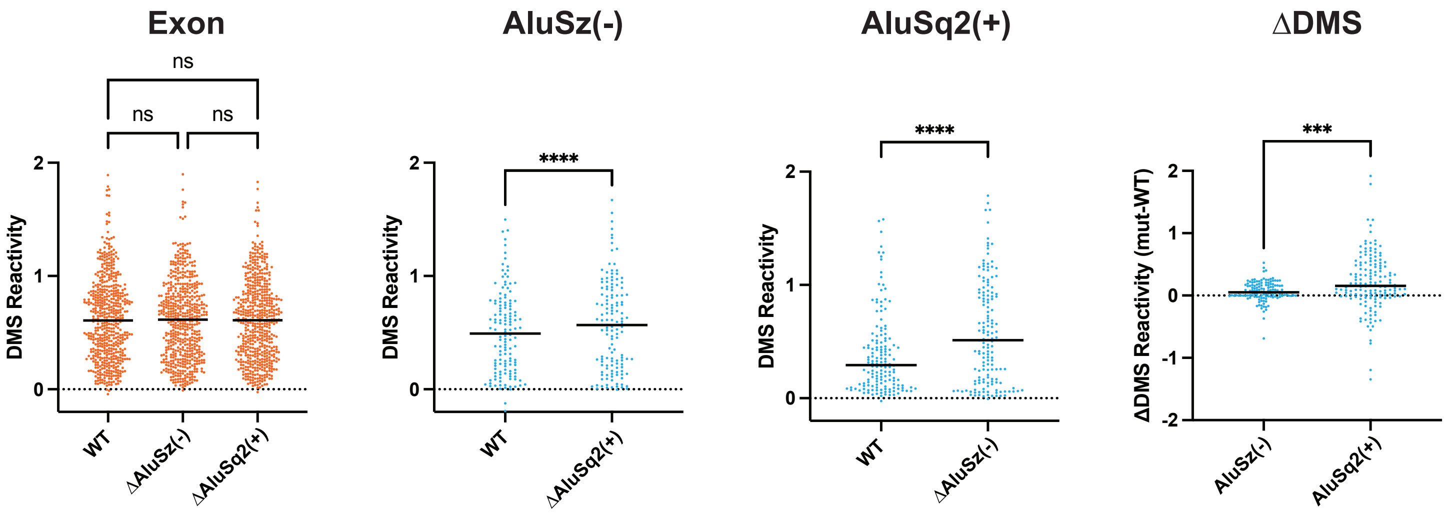

Supplementary Figure 4: DMS regional median reactivity. Points represent DMS reactivities of each nucleotide, with the median shown as a black line. Only adenosines and cytidines are analyzed. Significance was assessed with a one-way ANOVA for the Exon, a paired t-test for the AluSz(-) and AluSq2(+), and an unpaired t-test for ∆DMS. Significance: ns not significant, * $p < 0.05$, ** $p < 0.01$, *** $p < 0.001$, **** $p < 0.0001$

Supplementary Figure 5: DeltaShape

A AluSz(-)

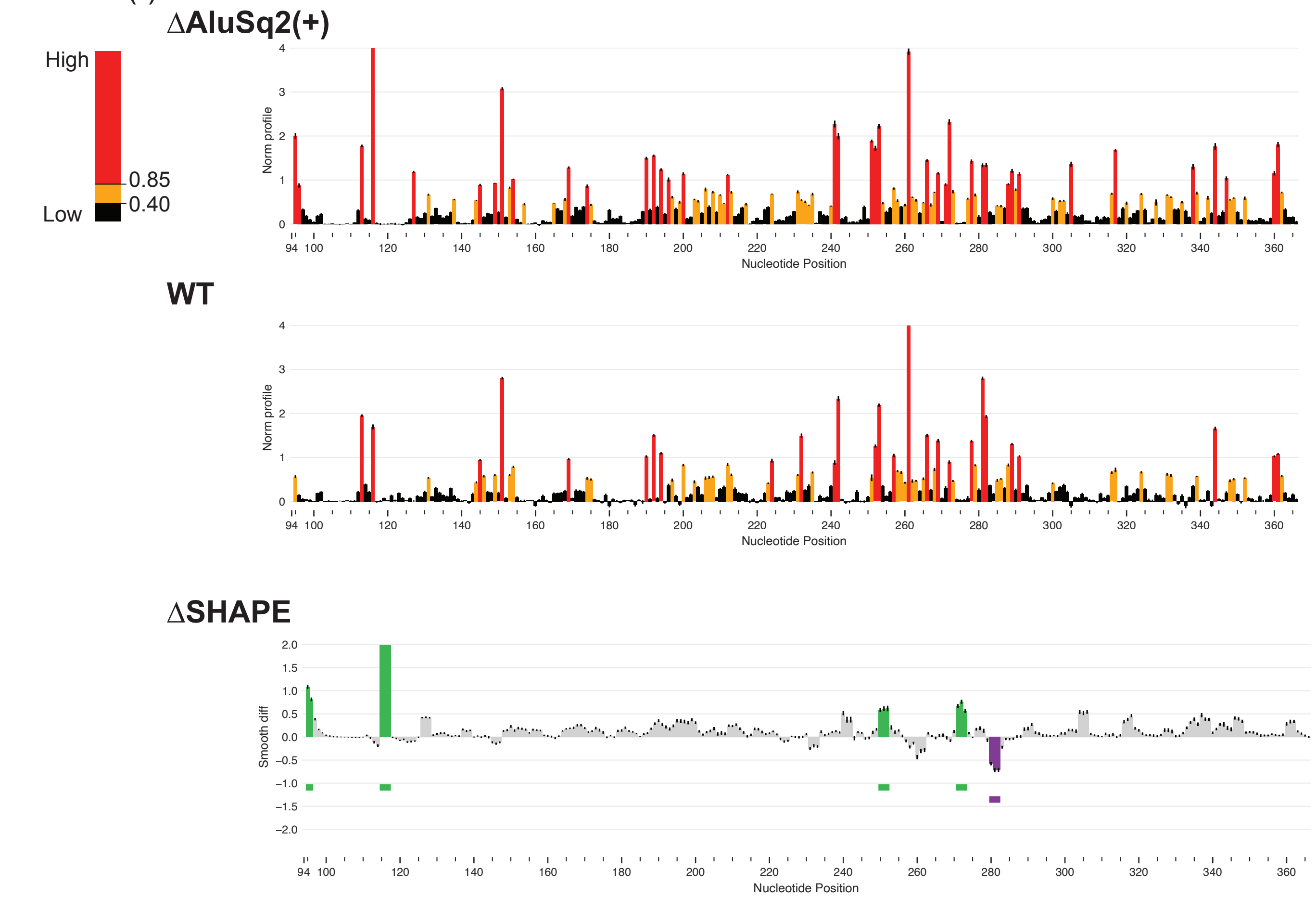

B AluSq2(+)

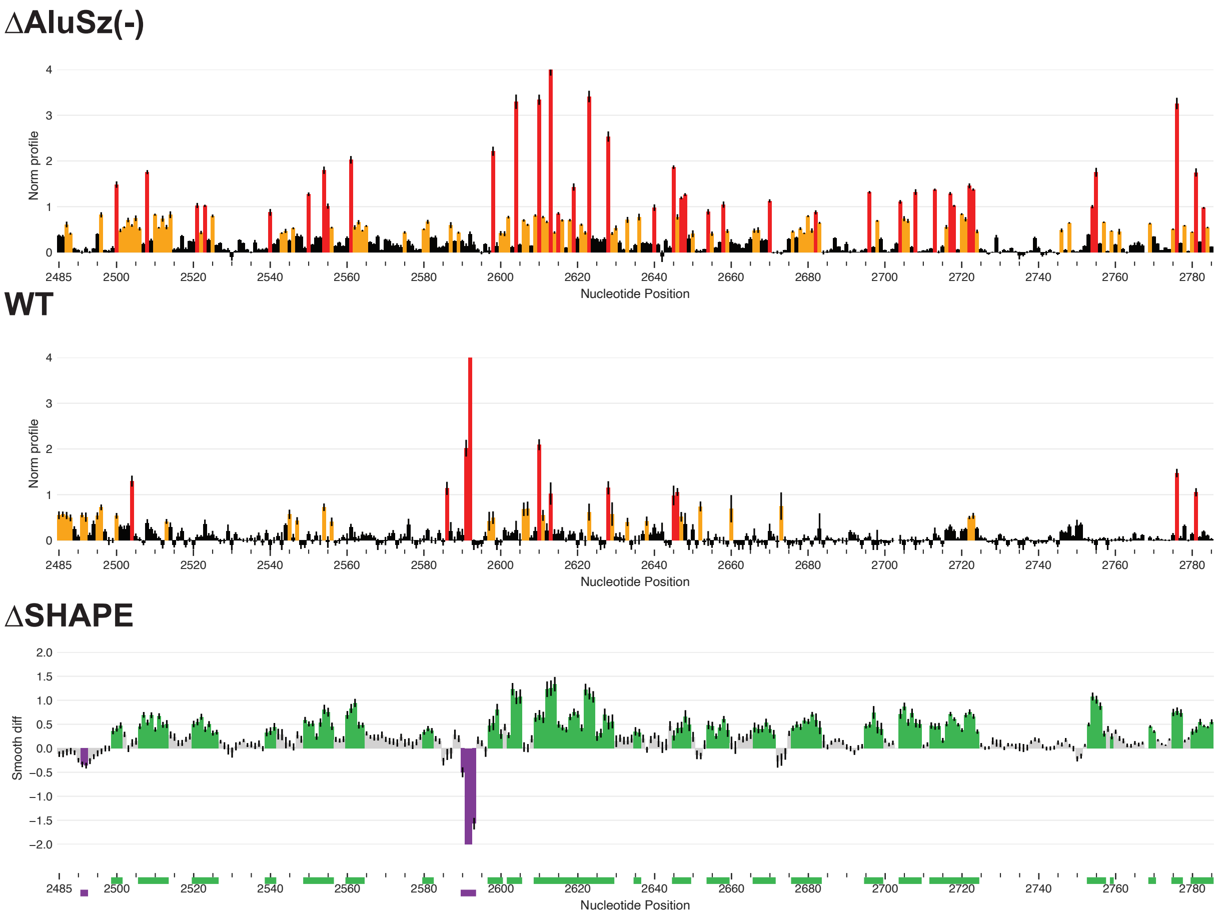

Supplementary Figure 5: ∆SHAPE Alu analysis **A.** ∆SHAPE analysis of AluSz(-). Top panel is the normalized SHAPE profile of AluSz(-) in the ∆AluSq2(+) context, whereas the middle panel is normalized SHAPE profile of AluSz in the wild-type context. Bottom panel is the difference between the two (∆SHAPE) across AluSz(-), (∆AluSq2(+) construct – WT construct), with statistical differences (as called by DeltaSHAPE analysis in RNAvigate) highlighted in color. Green indicates regions that have significantly increased SHAPE reactivity in the deletion construct, whereas purple indicates regions that have significantly decreased SHAPE reactivity in the deletion construct. Colored bars below the reactivity plots are highlighting the same regions. **B.** ∆SHAPE analysis of AluSq2(+). Top panel is the normalized SHAPE profile of AluSq2(+) in the ∆AluSz(-) context, whereas the middle panel is the normalized SHAPE profile of AluSq2 in the wild-type context. Bottom panel is the difference between the two (∆SHAPE) across AluSq2(+), (∆AluSz(-) construct – WT construct) , with statistical differences (as called by DeltaSHAPE analysis in RNAvigate) highlighted in color as in A.

Supplementary Figure 6: ΔAluSq2(+) and structure of AluSz(-) without its pairing partner

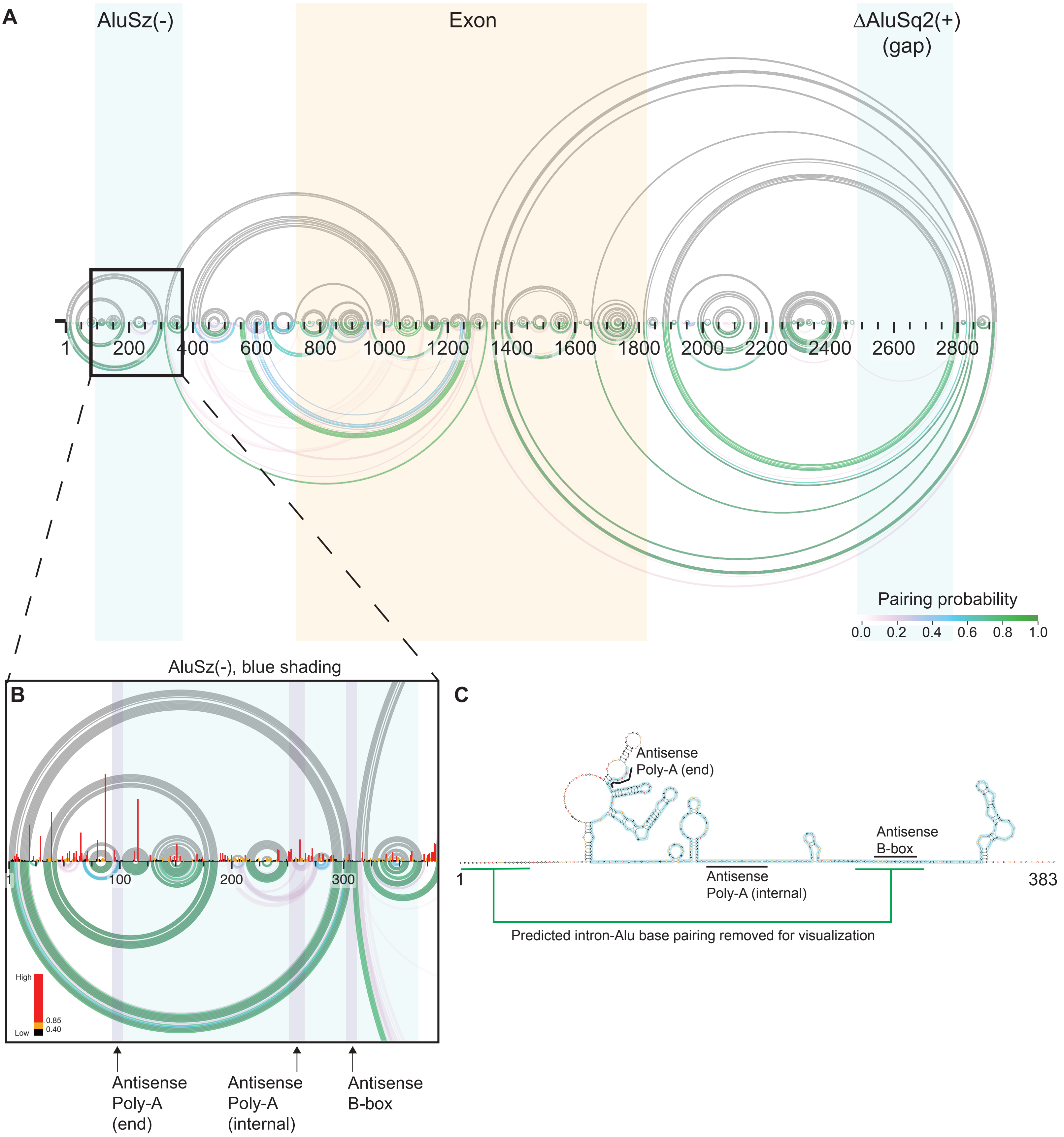

Supplementary Figure 6: SHAPE-informed AluSz(-) (ΔAluSq2(+)) structure **A.** SHAPE-informed arcplot structures of ΔAluSq2(+). Top (grey) arcs represent the minimum free energy structure, whereas the bottom arcs represent pairing probabilities. Blue blocks identify the Alu regions (gap indicates the deletion), whereas the orange block identifies the exon. **B.** Inset, zoomed in on the region including AluSz(-) (blue shading), with the normalized SHAPE profile. Alu element features are indicated by purple shading and annotated with arrows below. **C.** Secondary structure diagram of AluSz(-) and its flanking sequence. The Alu element is outlined in blue, and nucleotides are colored by normalized SHAPE reactivity. Alu features are annotated and denoted with a black line. Predicted intron-Alu base pairing was removed for improved visualization of the intra-Alu structure, denoted by the green line.

Supplementary Figure 7: ΔAluSz(-) and structure of AluSq2(+) without its pairing partner

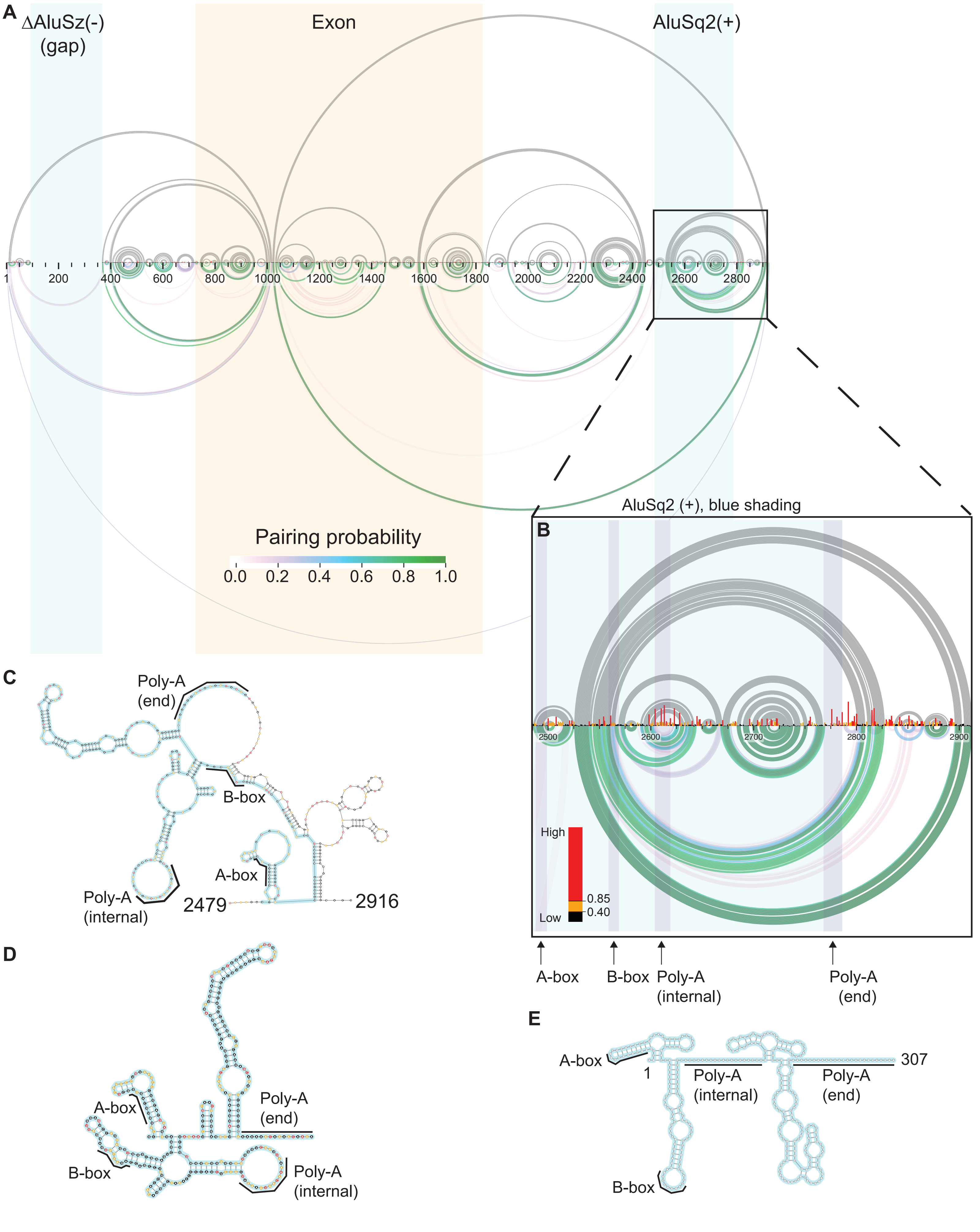

Supplementary Figure 7: SHAPE-informed AluSq2(+) (∆AluSz(-)) structure **A.** SHAPE-informed arcplot structures of ∆AluSz(-). Top (grey) arcs represent the minimum free energy structure, whereas the bottom arcs represent pairing probabilities. Blue blocks identify the Alu regions (gap indicates the deletion), whereas the orange block identifies the exon. **B.** Inset, zoomed in on the region including the AluSq2(+) (blue shading), with the normalized SHAPE profile. Alu element features are indicated by purple shading and annotated with arrows below. **C.** Secondary structure of AluSq2(+) in its sequence context. The Alu element is highlighted in blue and nucleotides are colored by normalized SHAPE reactivity. Alu features are annotated and denoted with a black line. **D.** Secondary structure of AluSq2(+) folded alone, with SHAPE data but without any sequence context. The Alu element is highlighted in blue and nucleotides are colored by normalized SHAPE reactivity. Alu features are annotated and denoted with a black line. **E.** Published secondary structure of an AluSx1(+) element (31), with common Alu features annotated. The Alu element is highlighted in blue and Alu features are annotated and denoted with a black line.

Supplementary Figure 8: Circularization Assay schematic and validation

A

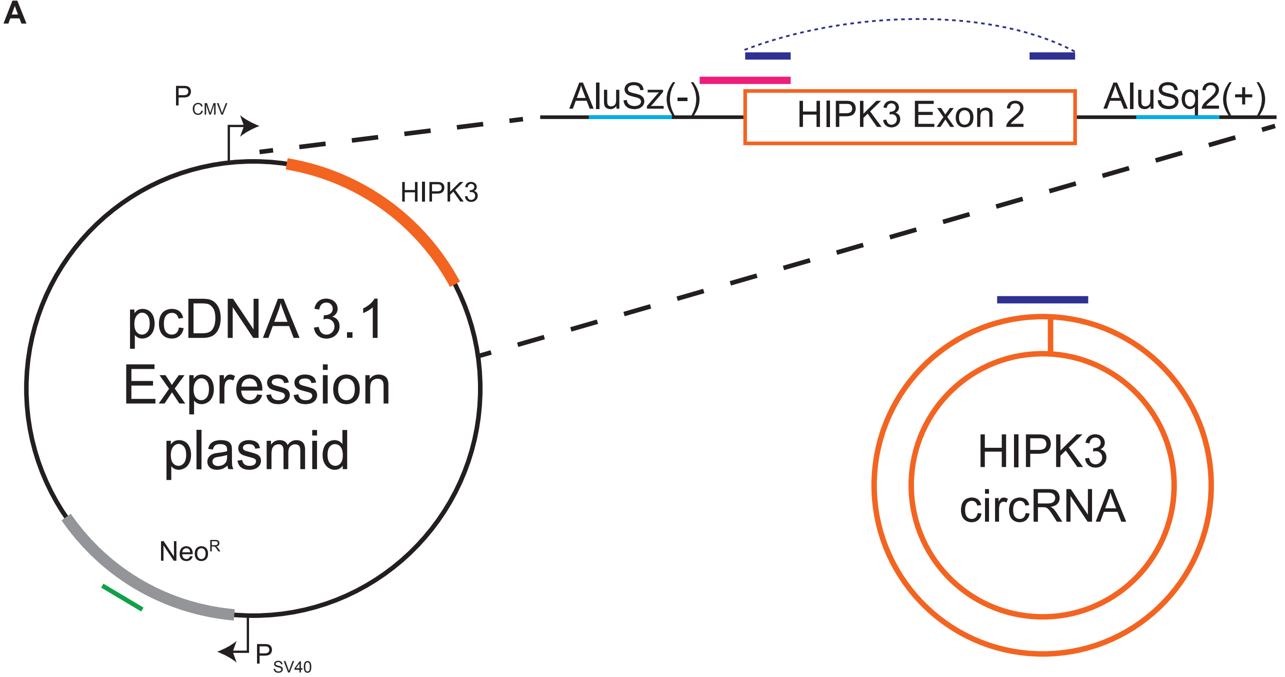

B

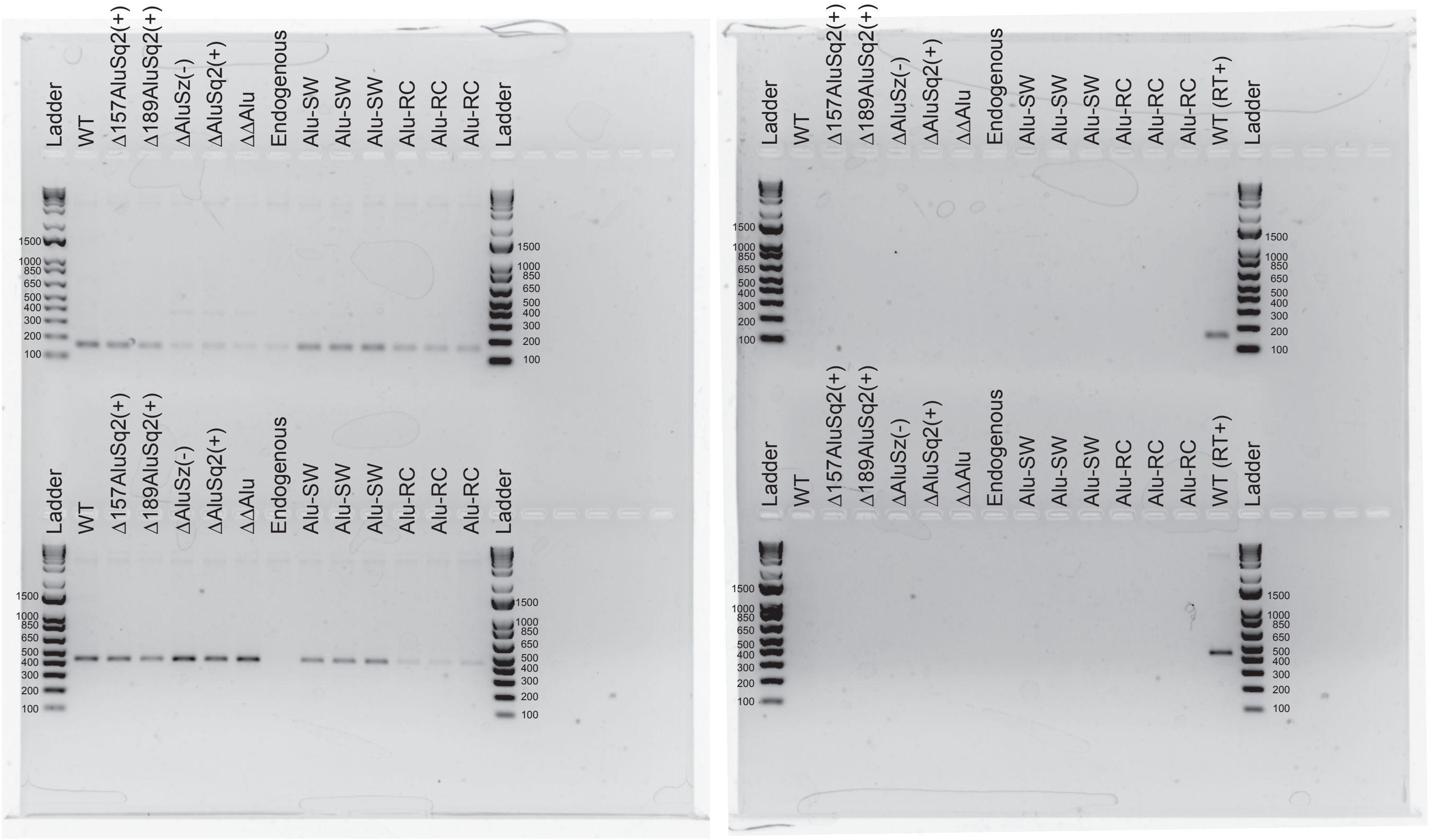

Supplementary Figure 8: Circularization Assay schematic and validation. **A.** The HIPK3 construct is expressed from a CMV promoter in the pcDNA 3.1 expression plasmid. One primer set specifically amplifies the circular form of HIPK3 exon 2 by priming outward from the exon in the linear depiction, and across the backsplice junction in the circRNA product, producing the dark blue amplicon. Another primer set specifically amplifies pre-mRNA by amplifying the intron-exon junction, producing the pink amplicon. Lastly, a control primer set amplifies the Neomycin Resistance gene on the pcDNA 3.1 plasmid, producing the green amplicon, for normalization to account for any variance in transfection efficiency. **B.** Agarose gel results of RT-PCR assays. Left gel contains RT-PCR samples, whereas the right gel shows controls without a reverse transcriptase (except for WT RT(+) positive control sample). Top half of each gel shows circularization product (155 bp), bottom half shows the pre-mRNA product (424 bp). “Endogenous” label is the background HeLa expression of HIPK3 with no plasmid transfected, whereas Alu-SW represents positionally swapped Alus and Alu-RC represents both Alus being reverse complemented in place.

Supplementary Figure 9: Circularization efficiency and regional SHAPE reactivity of rearrangement constructs.

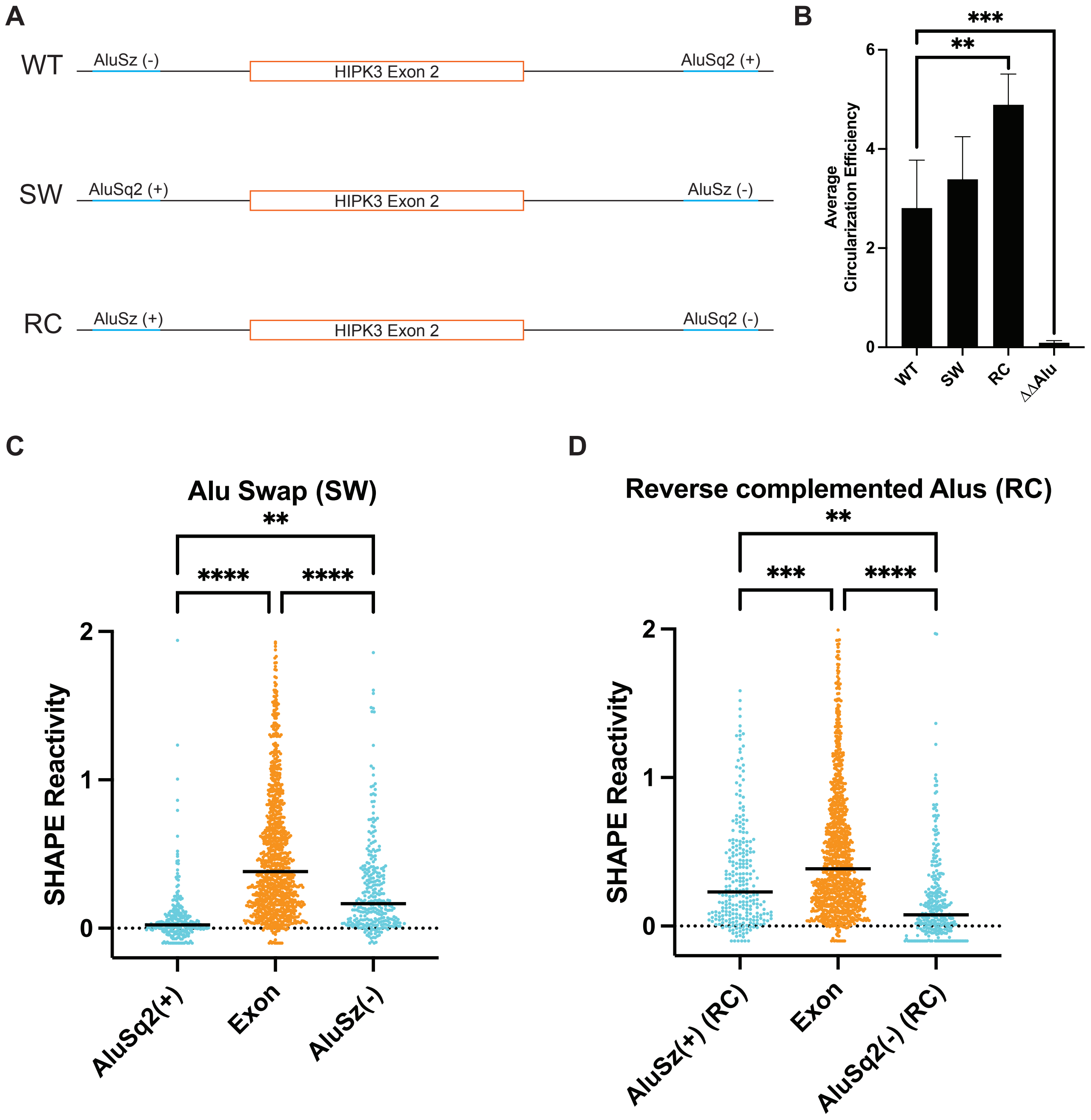

Supplementary Figure 9: Circularization efficiency and regional SHAPE reactivity of rearrangement constructs. **A.** Construct design illustrating the rearrangements of Alu elements in both position and orientation. **B.** Circularization efficiency as determined by RT-qPCR of wild type and rearrangement mutants, also showing ∆∆Alu as a control comparison for no circularization. Significance determined with an ordinary one-way ANOVA and Dunnett's multiple comparisons test. Significance throughout all figures: * p < 0.05, ** p < 0.01, *** p < 0.001, **** p < 0.0001 **C.** Median regional normalized SHAPE comparing each Alu (blue) and the exon (orange) in the swapped Alu construct. Regions are ordered on the x-axis in the order they appear in the construct, 5' to 3'. **D.** Regional reactivity of Alu elements and the exon in the reverse complement Alu construct.

Supplementary Figure 10: JuMP replicates

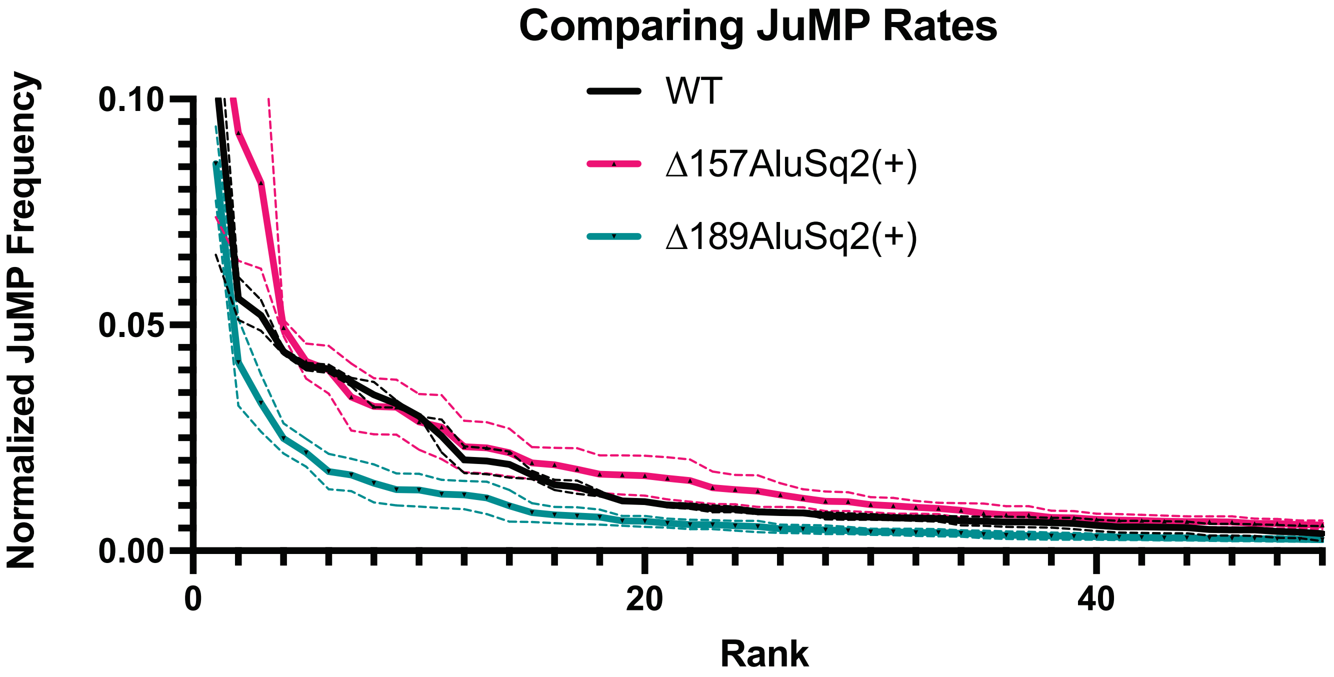

Supplementary Figure 10: JuMP replicate comparison. Solid lines show the normalized JuMP frequency of the highest frequency JuMP events for each construct (WT black, Δ157AluSq2(+) pink, Δ189AluSq2(+) teal). Dashed lines represent the range of two replicates. Rank denotes the order of JuMP events from the highest frequency to the lowest.
